# In vitro and in vivo analyses of eFAP: a novel FAP-targeting small molecule for radionuclide theranostics and other oncological interventions

**DOI:** 10.1186/s41181-024-00283-x

**Published:** 2024-07-29

**Authors:** Circe D. van der Heide, Hanyue Ma, Mark W.H. Hoorens, Joana D. Campeiro, Debra C. Stuurman, Corrina M.A. de Ridder, Yann Seimbille, Simone U. Dalm

**Affiliations:** 1 Department of Radiology and Nuclear Medicine, Erasmus MC University Medical Center Rotterdam, Rotterdam, 3015 GD The Netherlands; 2 Department of Experimental Urology, Erasmus Medical Center, University Medical Center Rotterdam, Rotterdam, 3015 GD The Netherlands; 3 Life Sciences Division, TRIUMF, Vancouver, BC V6T 2A3 Canada

**Keywords:** Cancer-associated fibroblast (CAF), Fibroblast activation protein (FAP), Radionuclide theranostics, Small molecule inhibitor, (4-quinolinoyl)-glycyl-2-cyanopyrrolidine (QCP)

## Abstract

**Background:**

Fibroblast activation protein (FAP), a transmembrane serine protease overexpressed by cancer-associated fibroblasts in the tumor stroma, is an interesting biomarker for targeted radionuclide theranostics. FAP-targeting radiotracers have demonstrated to be superior to [^18^F]FDG PET/CT in various solid cancers. However, these radiotracers have suboptimal tumor retention for targeted radionuclide therapy (TRT). We aimed to develop a novel FAP-targeting pharmacophore with improved pharmacokinetics by introducing a substitution at the 8-position of (4-quinolinoyl)-glycyl-2-cyanopyrrolidine, which allows for conjugation of a chelator, dye, or other payloads.

**Results:**

Here we showed the synthesis of DOTA-conjugated eFAP-6 and sulfo-Cyanine5-conjugated eFAP-7. After chemical characterization, the uptake and specificity of both tracers were determined on FAP-expressing cells. In vitro, [^111^In]In-eFAP-6 demonstrated a superior affinity and a more rapid, although slightly lower, peak uptake than gold standard [^111^In]In-FAPI-46. Confocal microscopy demonstrated a quick FAP-mediated internalization of eFAP-7. Studies with HT1080-huFAP xenografted mice confirmed a more rapid uptake of [^177^Lu]Lu-eFAP-6 vs. [^177^Lu]Lu-FAPI-46. However, tumor retention at 24 h post injection of [^177^Lu]Lu-eFAP-6 was lower than that of [^177^Lu]Lu-FAPI-46, hereby currently limiting its use for TRT.

**Conclusion:**

The superior affinity and faster tumor accumulation of eFAP-6 over FAPI-46 makes it a suitable compound for radionuclide imaging. After further optimization, the eFAP series has great potential for various oncological interventions, including fluorescent-guided surgery and effective targeted radionuclide theranostics.

**Supplementary Information:**

The online version contains supplementary material available at 10.1186/s41181-024-00283-x.

## Background

Cancer-associated fibroblasts (CAFs) are one of the most predominant components in the tumor microenvironment (TME) of solid cancers. CAFs support tumor growth, promote metastasis, and can induce therapy resistance (Cirri and Chiarugi 2012; De et al. 2021; Saw et al. 2022; Koustoulidou et al. 2021). For these reasons, CAFs have emerged as an appealing target for anti-cancer therapies, and various CAF-targeted treatment strategies have been developed. Many of these strategies target the fibroblast activation protein (FAP), which is almost exclusively expressed on CAFs, although FAP-expression by fibroblasts in inflammatory disease and by some types of tumor cells is also reported (Yazbeck et al. 2018; Liu et al. 2019). FAP-expressing CAFs have been found in over 90% of epithelial carcinomas (Garin-Chesa et al. 1990; Mhawech-Fauceglia et al. 2015), and high FAP expression is associated with poor prognosis and worse treatment outcomes in multiple cancers (Lo et al. 2017; van der Heide & Dalm 2022; Saigusa et al. 2011; Moreno-Ruiz et al. 2021). Thus, successful development of FAP-targeted anti-cancer interventions can be beneficial for a large number of solid cancers.

Targeted radionuclide therapy (TRT) and radionuclide imaging are already successfully applied in the clinic for the detection and treatment of various cancer types (e.g., metastatic prostate cancer and neuro-endocrine tumors) (Hennrich and Eder 2022; Strosberg et al. 2017). For these radionuclide interventions, a radiopharmaceutical is administered that binds to a cancer-specific biomarker for selective delivery of a radioisotope, which either has properties suitable for PET/SPECT imaging, or for the delivery of cytotoxic radiation to the tumor cells. Recently, various FAP-targeting radiotracers have been developed and tested, including small molecule radiotracers FAPI-04 and FAPI-46, which are widely evaluated in several preclinical and clinical studies with promising results (Loktev et al. 2019; Zhao et al. 2022; Fendler et al. 2023). For example, a study by Giesel et al., (2021) demonstrated superior tumor-to-background ratios (TBR) in PET/CT scans with [^68^Ga]Ga-FAPI-04 compared to conventional [^18^F]FDG PET/CT, especially for solid tumors with high physiological FDG uptake (Giesel et al. 2021). Despite the successes demonstrated in imaging studies, most of the currently available FAP-targeting radiotracers demonstrate a suboptimal tumor retention time in preclinical and clinical studies (Loktev et al. 2019; Kuyumcu et al. 2021; Ballal et al. 2021; Sun et al. 2022). For more effective TRT, improved tumor retention of the radiotracer is necessary, to increase the radiation dose to which the tumor cells are exposed. For example, the FAP-binding cyclic peptide FAP-2286 has demonstrated that enhanced tumor retention can improve therapeutic outcomes in tumor-bearing mice (Zboralski et al. 2022).

**Fig. 1:**
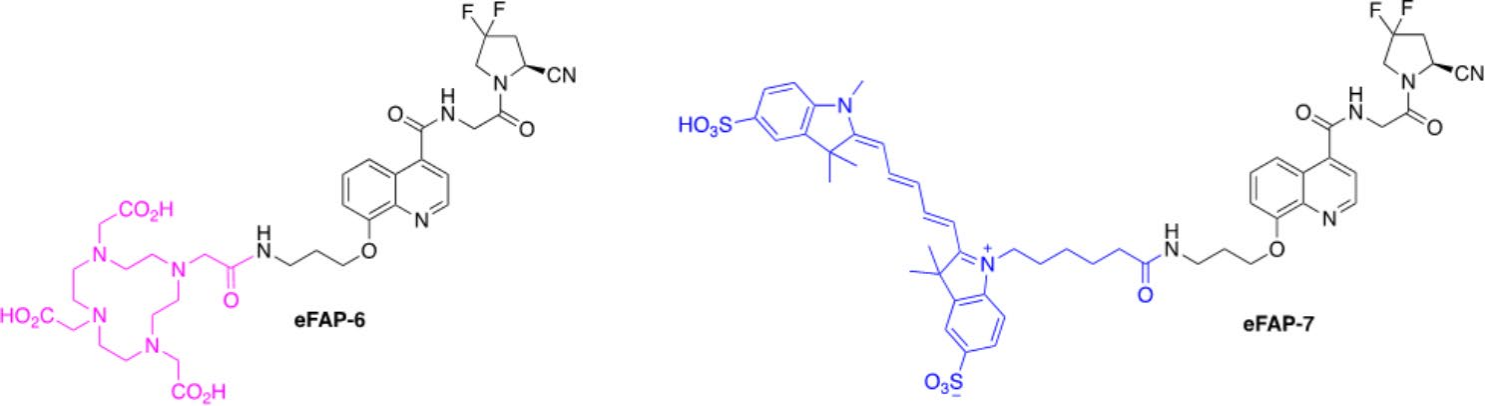
Chemical structures of the 8-QCP-based FAP inhibitors eFAP-6 and eFAP-7, containing a DOTA chelator (pink) and a sCy5 dye (blue), respectively

FAPI-04 and FAPI-46, as well as many other FAP-targeting small molecule radiotracers, are based on the *N*-(4-quinolinoyl)-Gly-(2-cyanopyrrolidine) scaffold of the FAP inhibitor UAMC-1110 introduced by Jansen et al. (Jansen et al. 2013). Over time the development of derivatives of UAMC-1110 expanded and many new FAP-targeting small molecules were reported in literature (Mori et al. 2023; Moon et al. 2020). Although some of these novel FAP-targeting compounds demonstrated durable tumor accumulation (e.g., OncoFAP) (Millul et al. 2021), the modifications have not significantly improved the tumor retention time. This prompted us to investigate whether substitution at the 8-position of the quinoline ring via an ether linkage could result in a pharmacophore with improved pharmacokinetic properties for FAP-targeting radionuclide theranostics, without affecting FAP affinity. Additionally, the presence of a primary amine enables the conjugation of payloads, such as radionuclide chelators or fluorophores. This could result in a wide range of applications, such as molecular imaging, radionuclide therapy, and fluorescence-guided surgery (FGS). Here, we describe the development, as well as the in vitro and in vivo evaluation of two of our developed FAP-targeting compounds; the radiotracer eFAP-6 conjugated with a DOTA chelator, suitable for labeling with indium-111 for SPECT imaging and lutetium-177 for future TRT studies, and the imaging probe eFAP-7 conjugated with a sulfo-Cyanine5 (sCy5) for fluorescent imaging (Fig. 1).

## Results

### Chemistry and radiochemistry

**Scheme 1:**
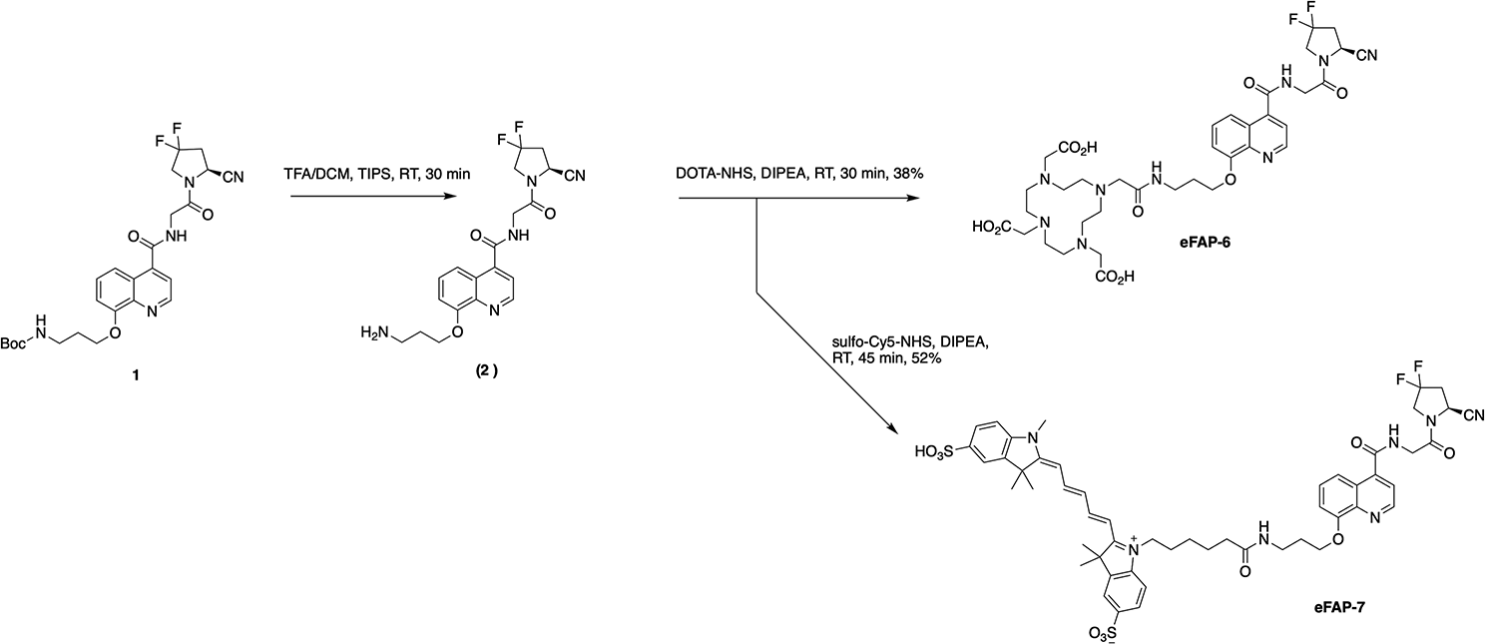
Chemical synthesis of the FAP inhibitors eFAP-6 and eFAP-7 from 8-QCP **(1)**

The synthesis of the FAP inhibitors eFAP-6 and eFAP-7 based on (4-quinolinoyl)-glycyl-2-cyanopyrrolidine substitution at the 8-position (8-QCP) is presented in Scheme 1. The starting material **1**, resulting from the etherification of 8-QCP with a *tert*-butyloxycarbonyl (Boc) protected propylamine, was prepared according to an adapted protocol previously reported by Lindner et al. (Lindner et al. 2018). Then, the DOTA-conjugated precursor eFAP-6 and the sCy5 equipped probe eFAP-7 were prepared in two steps; consisting of removal of the Boc protecting group from **1** in acidic conditions, followed by the conjugation of DOTA-NHS ester or sCy5-NHS ester on the primary amine to give eFAP-6 and eFAP-7 with a yield of 38% and 52%, respectively. Radiolabeling of eFAP-6 with 111-indium and 177-lutetium was performed successfully with high radiochemical yield and purity (> 95%). Detailed information on the synthesis, NMR, LC chromatograms, and mass spectra is provided in the Supplementary Materials (Figures S1-S7).

### Stability and lipophilicity

The stability of [^111^In]In-eFAP-6 and [^177^Lu]Lu-eFAP-6 was assessed by radio-high-performance liquid chromatography (HPLC) after incubation in PBS, mouse serum (MS), and human serum (HS) for 1, 4, and 24 h (Table 1, Figure S8-S13). Both [^111^In]In-eFAP-6 and [^177^Lu]Lu-eFAP-6 displayed high stability in all media for at least 24 h since less than 10% degradation occurred for [^111^In]In-eFAP-6, and impressively even less than 5% degradation for [^177^Lu]Lu-eFAP-6 in PBS, MS, and HS up to 24 h of incubation (Table 1). eFAP-7 also demonstrated high stability when incubated for 24 h in PBS (99.1 ± 0.1%). However, in both HS and MS increasing degradation was observed over time, resulting in 76.2 ± 7.1% (MS) and 85.5 ± 2.1% (HS) intact eFAP-7 at 24 h (Table 1, Figure S14-S16). Next lipophilicity (LogD_7.4_) was determined. [^111^In]In-eFAP-6 showed pronounced hydrophilic properties with a logD_7.4_ value of -3.90 ± 0.03. However, eFAP-7 demonstrated a relatively increased lipophilic character (logD_7.4_ = -0.94 ± 0.26) (Table 1).

**Table 1:** Lipophilicity (LogD_7.4_) and stability studies in PBS, mouse and human serum after 24 h

Compound	LogD_7.4_	Stability (% ± SD^a^)								
		PBS			Mouse serum			Human serum		
		1 h	4 h	24 h	1 h	4 h	24 h	1 h	4 h	24 h
[^111^In]In-eFAP-6	-3.90 ± 0.03	93.2 ± 0.5%	93.1 ± 0.6%	92.3 ± 0.6%	92.1 ± 1.2%	92.1 ± 1.4%	91.0 ± 0.9%	91.2 ± 0.1%	91.3 ± 0.7%	90.4 ± 0.8%
[^177^Lu]Lu-eFAP-6	na	97.6 ± 1.1%	97.3 ± 0.4%	94.9 ± 0.6%	99.3 ± 1.2%	99.5 ± 0.5%	98.1 ± 0.4%	99.5 ± 0.0%	99.5 ± 0.0%	98.1 ± 0.3%
eFAP-7	-0.94 ± 0.26	99.5 ± 0.5%	99.2 ± 0.7%	99.1 ± 0.1%	85.0 ± 3.3%	83.2 ± 4.1%	76.2 ± 7.1%	87.6 ± 0.9%	87.9 ± 2.3%	85.5 ± 2.1%

^a^ Results are expressed as a percentage (%) of intact labeled ligand after incubation at 37 °C

na = not available. PBS = phosphate-buffered saline

### Affinity for recombinant huFAP, muFAP, PREP, and DPP4

**Fig. 2:**
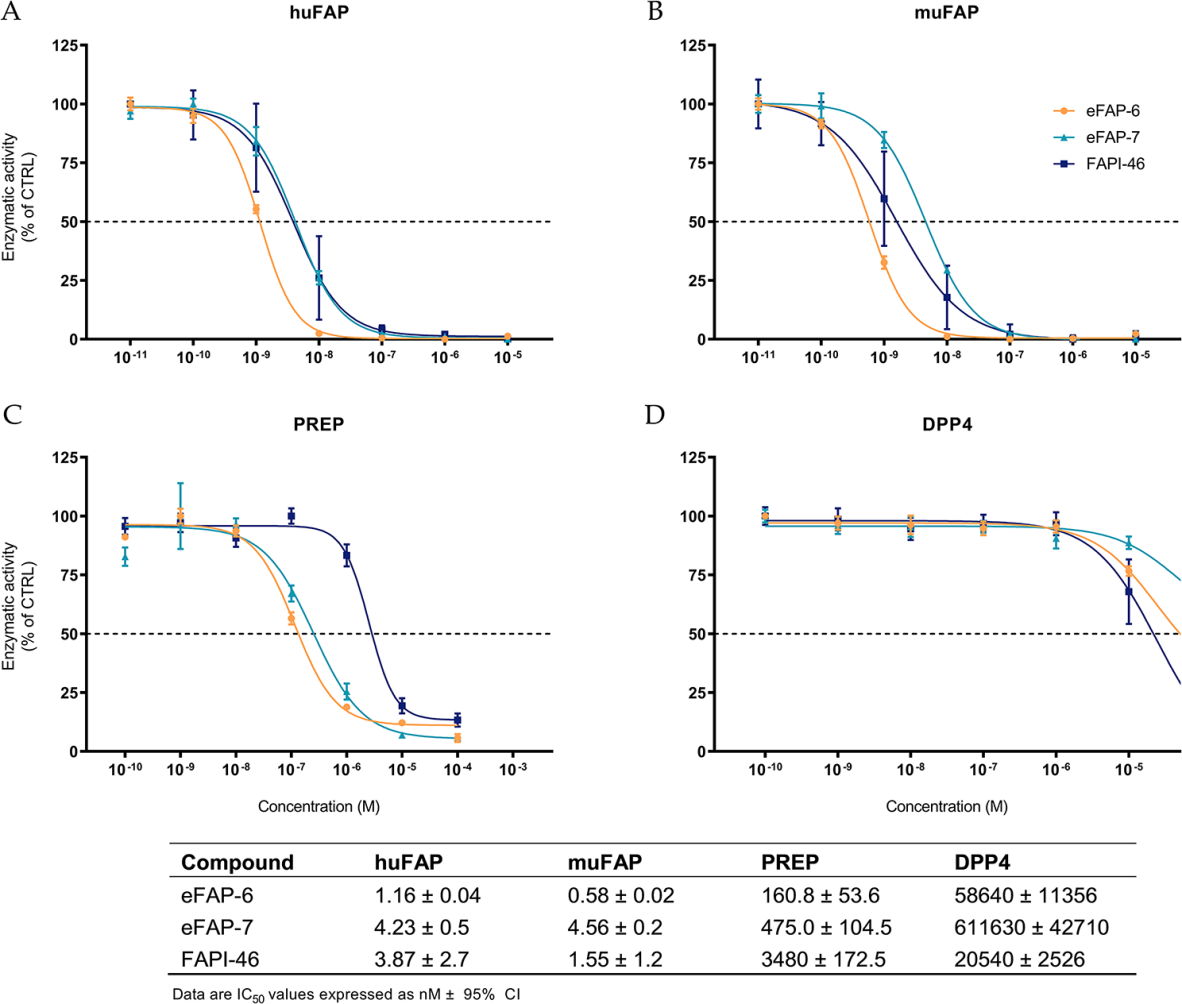
Enzymatic activity inhibition of recombinant (**A**) huFAP (*n* = 3), (**B**) muFAP (*n* = 3), (**C**) PREP (*n* = 3), and (**D**) DPP4 (*n* = 3) incubated with eFAP-6, eFAP-7, and FAPI-46. Data are expressed as mean ± SD

The affinity of eFAP-6 and eFAP-7 for human (hu)FAP and murine (mu)FAP was first determined in a recombinant enzymatic assay. Both FAP inhibitors exhibited binding affinity to huFAP and muFAP in the nanomolar range, comparable to that of FAPI-46 (IC_50 huFAP_ = 3.87 ± 2.7 nM, IC_50 muFAP_ = 1.55 ± 1.2 nM). Of the three compounds, eFAP-6 showed the highest inhibitory activity for both FAP proteins (IC_50 huFAP_ = 1.16 ± 0.04 nM, IC_50 muFAP_ = 0.58 ± 0.02 nM) (Fig. 2A, B). The sCy5-conjugated eFAP-7 (IC_50 huFAP_ = 4.23 ± 0.5 nM, IC_50 muFAP_ = 4.56 ± 0.2 nM) had a somewhat lower affinity than FAPI-46. Additionally, to evaluate the selectivity of our compounds over proteins that belong to the same family, enzymatic assays with prolyl oligopeptidase (PREP) and dipeptidyl peptidase IV (DPP4) were carried out. A low affinity of eFAP-6 and eFAP-7 for PREP was found (IC_50 PREP_ = 160.8 ± 53.6 and 475.0 ± 104.5 nM, respectively) (Fig. 2C), and the IC_50_ values of eFAP-6 and eFAP-7 for DPP4 were even higher, in the millimolar range (Fig. 2D). This resulted in high PREP/huFAP and DPP4/huFAP selectivity indexes (SI > 100) (Table 2).

**Table 2:** Selectivity indexes (SI)

Compound	SI PREP/huFAP	SI DPP4/huFAP
eFAP-6	138.6	50551.7
eFAP-7	112.3	144593.4
FAPI-46	899.2	5307.5

### [^111^In]In-FAPI-46 displacement assay on FAP-expressing cells

**Fig. 3:**
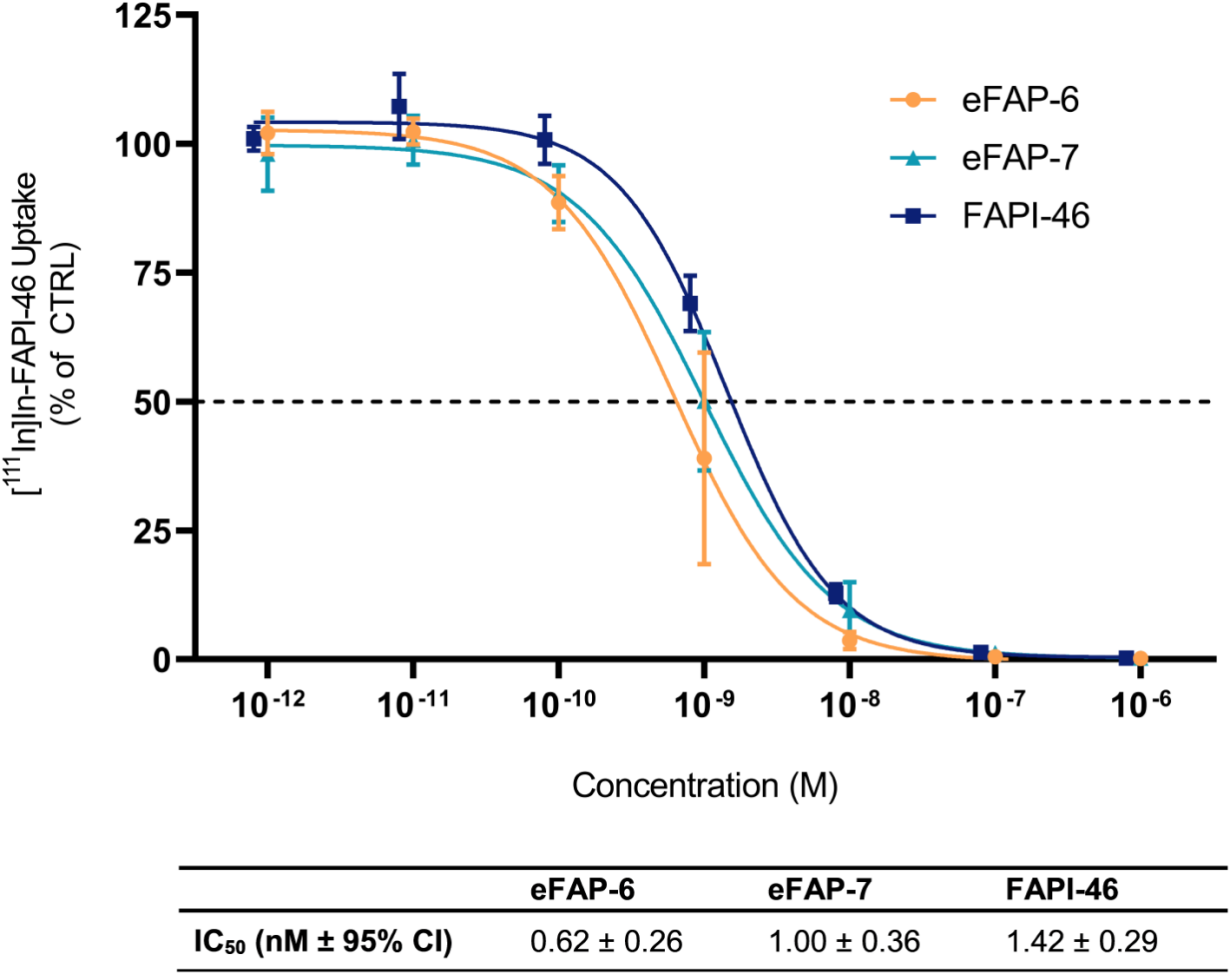
HT1080-huFAP cells incubated with increasing concentrations of unlabeled eFAP-6, eFAP-7, and FAPI-46 in a displacement assay with 10^− 9^ M [^111^In]In-FAPI-46 (*n* = 3). Data are expressed as mean ± SD

Next, the affinity of the compounds was determined on HT1080-huFAP cells in a displacement assay using [^111^In]In-FAPI-46. Both unlabeled eFAP-6 and eFAP-7 demonstrated potent displacement of [^111^In]In-FAPI-46, resulting in an IC_50_ of 0.62 ± 0.26 nM and 1.0 ± 0.36 nM, respectively. Impressively, these IC_50_ values were lower than that of FAPI-46 (IC_50_ = 1.43 ± 0.29 nM) (Fig. 3).

### Radiotracer uptake in FAP-expressing cells

**Fig. 4:**
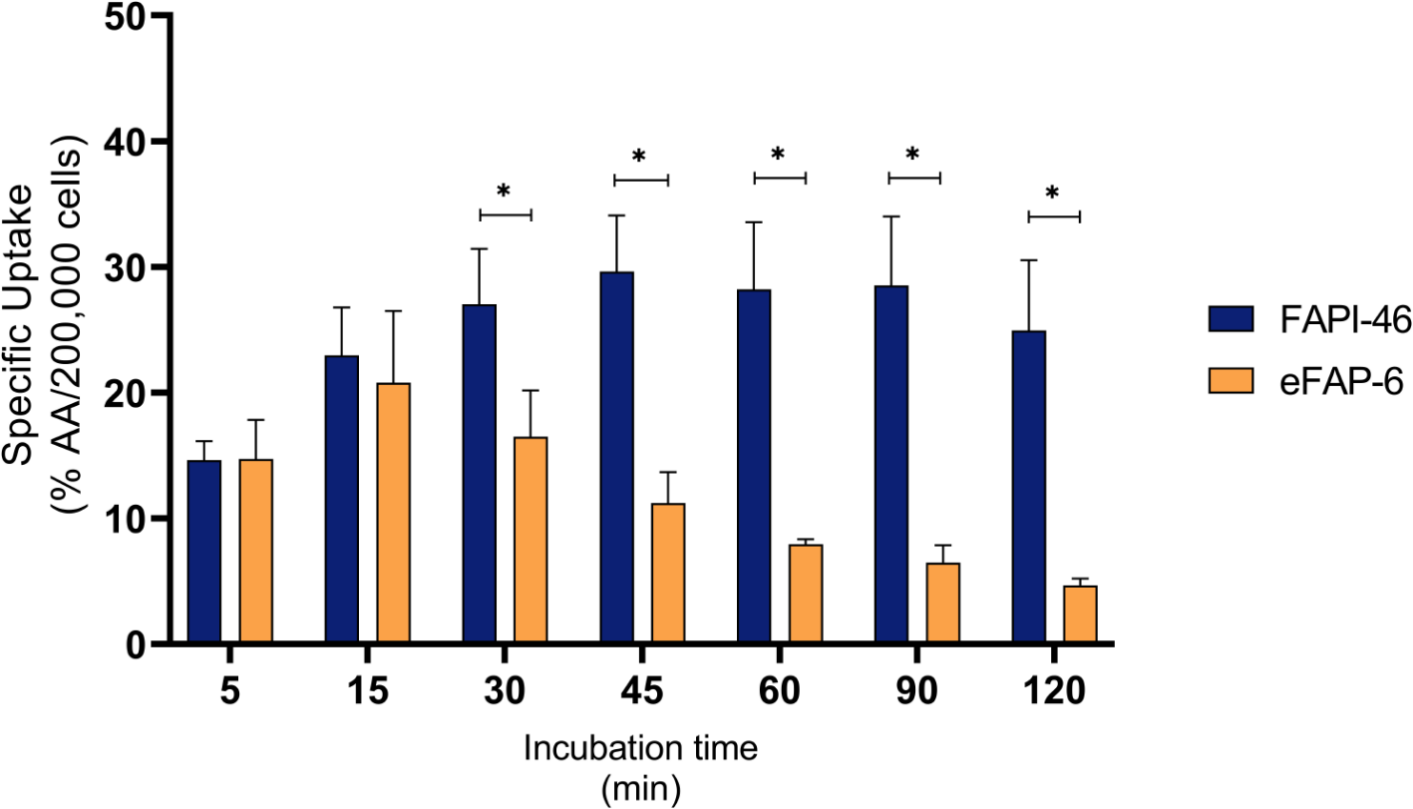
Uptake of [^111^In]In-FAPI-46 (*n* = 4) and [^111^In]In-eFAP6 (*n* = 3) by HT1080-huFAP cells at different time points (5–120 min) expressed as % AA/200,000 cells. Data are expressed as mean ± SD, *p < 0.05

First, the specificity of [^111^In]In-FAPI-46 and [^111^In]In-eFAP-6 uptake by HT1080-huFAP cells was confirmed by co-incubation with an excess of FAP-inhibitor UAMC-1110, which resulted in negligible uptake (Figure S17). Next, the uptake of [^111^In]In-eFAP-6 and [^111^In]In-FAPI-46 at increasing time points (5–120 min) was determined. [^111^In]In-eFAP-6 demonstrated a peak uptake of 20.8% AA/200,000 cells after 15 min incubation, whereas [^111^In]In-FAPI-46 reached a higher peak uptake of 29.6% AA/200,000 cells, after 45 min. Surprisingly, incubating the cells for more than 15 min with [^111^In]In-eFAP-6, resulted in a decreased uptake (Fig. 4B). This pattern was not observed for [^111^In]In-FAPI-46, for which the uptake seemingly reached a plateau between 45 and 120 min incubation (Fig. 4A). This resulted in a significantly higher uptake of FAPI-46 between 30 and 120 min incubation (Fig. 4C).

### Confocal microscopy of eFAP-7 on FAP-expressing cells

**Fig. 5:**
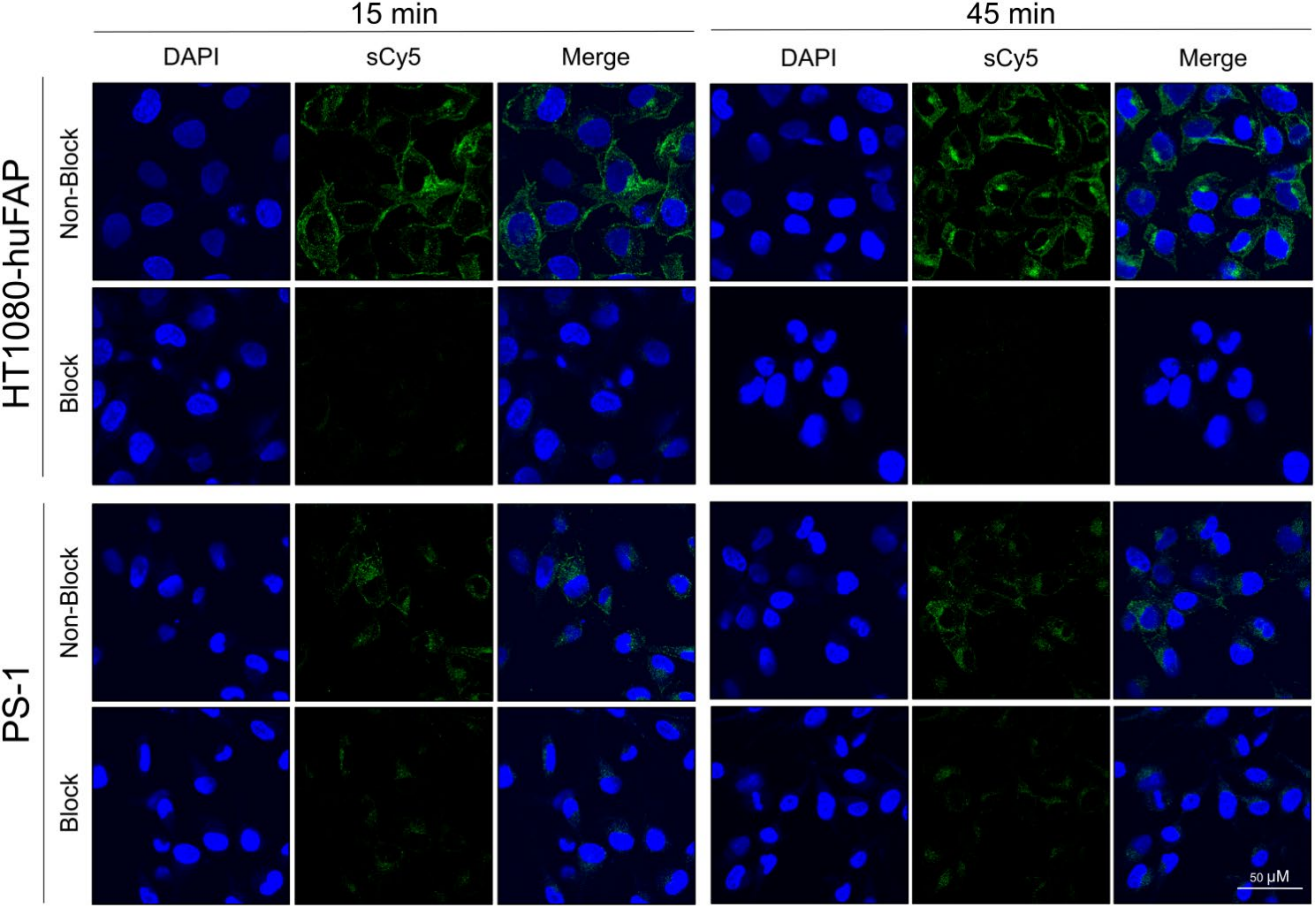
eFAP-7 uptake HT1080-huFAP cells, PS-1 cells, and HT1080-WT cells demonstrating the DAPI channel (blue), the sCy5 (eFAP-7) channel (green), and the overlay. Magnification is equal for all images. sCy5 = sulfo-Cyanine5

At 15 and 45 min of incubation with eFAP-7, a clear signal was visible in the HT1080-huFAP cells, with the highest signal at 45 min (Fig. 5). The uptake of eFAP-7 in PS-1 cells is visibly lower than for HT1080-huFAP at both time points, which is in line with the lower expression of FAP for this cell line. The uptake of eFAP-7 was specific for both cell lines, as there was almost no visible signal when the tracer was incubated simultaneously with an excess of UAMC-1110 (Fig. 5). To further confirm that the observed sCy5 signal is the result of FAP-mediated internalization of eFAP-7, HT1080-WT cells were also incubated with eFAP-7 and no uptake was observed (Figure S18).

### In vivo biodistribution studies

**Fig. 6:**
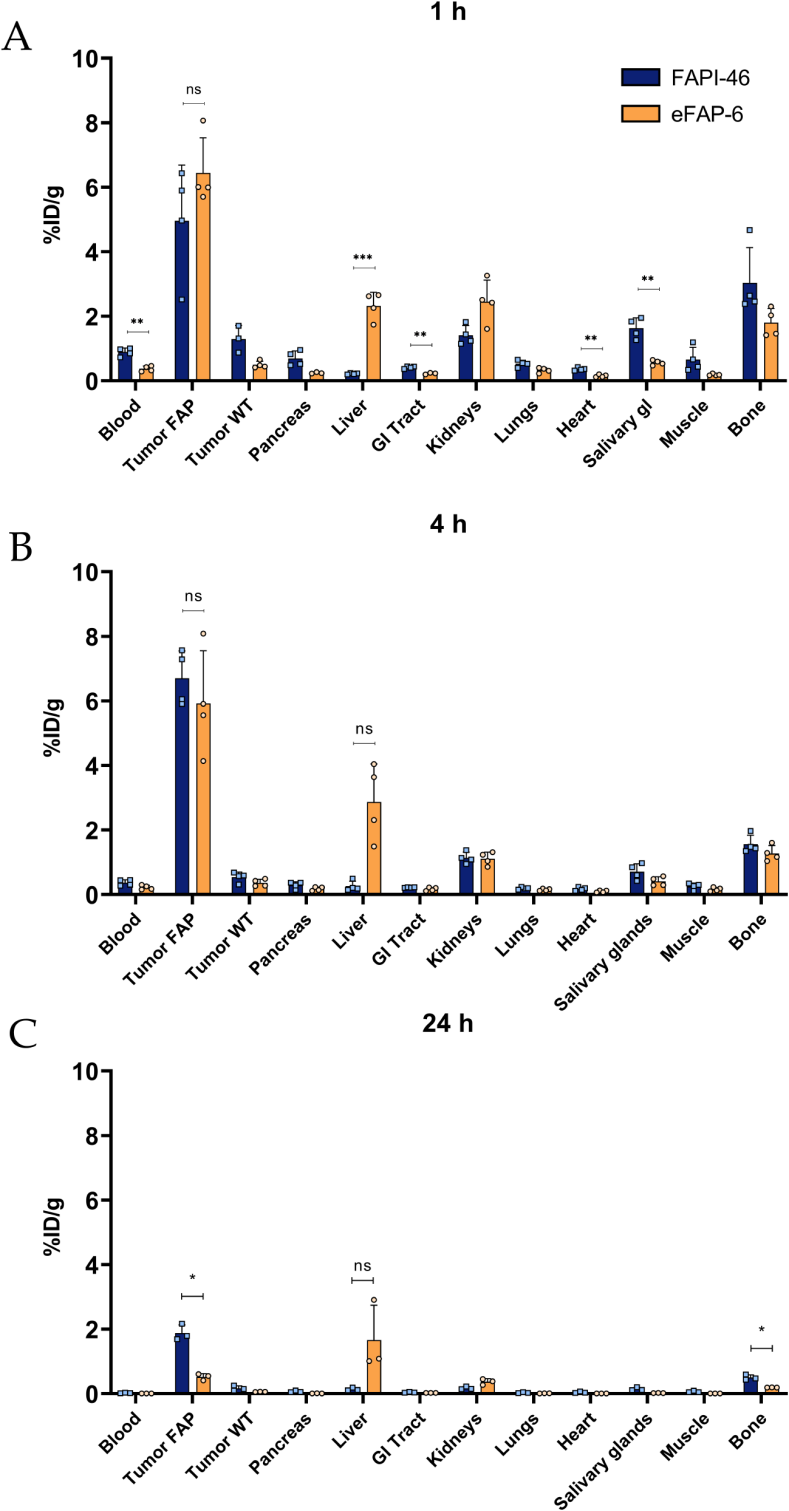
In vivo biodistribution studies. (**A**) 1, (**B**) 4, and (**C**) 24 h p.i. with [^177^Lu]Lu-FAPI-46 or [^177^Lu]Lu-eFAP-6.Data are expressed as mean ± SD with *n* = 3/4 per group. *p < 0.05 **p < 0.01 ***p < 0.001. ns = non-significant, GI Tract = Gastro-intestinal tract, WT = Wild type, FAP = Fibroblast activation protein

In line with in vitro results, the in vivo studies using HT1080-huFAP and HT1080-WT xenografted animals, demonstrated high and specific uptake of both [^177^Lu]Lu-FAPI-46 and [^177^Lu]Lu-eFAP-6 in the FAP-positive tumors. However, a difference in pharmacokinetics was observed; a more rapid uptake of [^177^Lu]Lu-eFAP-6 in the FAP-positive tumor compared to [^177^Lu]Lu-FAPI-46 was measured (6.4 ± 1.1% ID/g and 5.0 ± 1.7% ID/g 1 h post injection (p.i.), respectively) (Fig. 6A, Table S1). Moreover, the tumor uptake of [^177^Lu]Lu-FAPI-46 increased over time with the peak uptake being observed at 4 h p.i. (6.7 ± 0.9% ID/g), while [^177^Lu]Lu-eFAP-6 uptake at 4 h p.i. was 5.9 ± 1.6% ID/g, slightly lower than that observed at 1 h p.i. (Fig. 6B, Table S1). At 24 h p.i. [^177^Lu]Lu-eFAP-6 was almost completely washed out of the FAP-positive tumor, with only 0.5 ± 0.1% ID/g remaining, and while at 24 h p.i. the [^177^Lu]Lu-FAPI-46 uptake has also drastically decreased, it was significantly higher (1.9 ± 0.3% ID/g, *p* < 0.05) (Fig. 6C, Table S1).

**Fig. 7:**
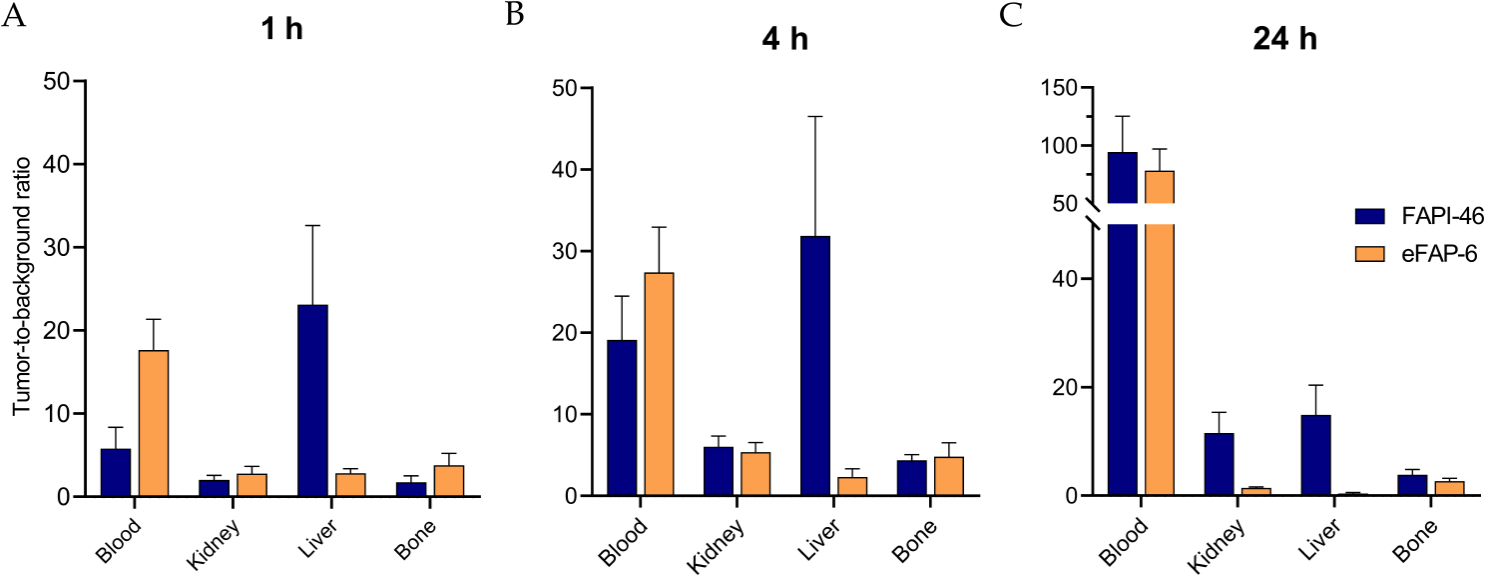
Tumor-to-background ratios of [^177^Lu]Lu-FAPI-46 and [^177^Lu]Lu-eFAP-6 based on the ex vivo biodistribution at (**A**) 1, (**B**) 4, and (**C**) 24 h p.i. (*n* = 3/4 per tracer). Data are expressed as mean ± SD

Regarding uptake in background organs, [^177^Lu]Lu-eFAP-6 demonstrated a higher accumulation in the liver; however, this was only significant at 1 h p.i. (2.3 ± 0.4% vs. 0.2 ± 0.02% ID/g, *p* < 0.001, Fig. 6A). In line with this, tumor-to-liver ratios of [^177^Lu]Lu-eFAP-6 were lower than that of [^177^Lu]Lu-FAPI-46 at all three time points (Fig. 7). The measured radioactivity in the kidneys after [^177^Lu]Lu-eFAP-6 injection was 2.5 ± 0.8, 1.1 ± 0.2, and 0.4 ± 0.1%ID/g compared to 1.4 ± 0.3, 1.1 ± 0.2, and 0.2 ± 0.1%ID/g for [^177^Lu]Lu-FAPI-46 at 1, 4, and 24 h p.i., respectively (Fig. 6). This resulted in similar tumor-to-kidney ratios for both radiotracers at 1 and 4 h p.i. (Fig. 7A, B). [^177^Lu]Lu-eFAP-6 demonstrated somewhat higher TBR ratios than [^177^Lu]Lu-FAPI-46 for the blood and bone at both 1 and 4 h (Fig. 7A, B). Overall, the radioactivity measured in the blood and other non-FAP expressing background organs was lower for [^177^Lu]Lu-eFAP-6 than for [^177^Lu]Lu-FAPI-46; however, this difference is only significant for the blood, GI tract, heart, and salivary glands at 1 h p.i. (*p* < 0.01, Fig. 6A), and for the bone at 24 h p.i. (*p* < 0.05)(Fig. 6C). The tumor-to-blood ratios were in favor of [^177^Lu]Lu-eFAP-6 for 1 and 4 h p.i., but this shifted at 24 h p.i. due to the higher tumor uptake of [^177^Lu]Lu-FAPI-46 at this time point (Fig. 7).

## Discussion

Current FAP-targeting small molecules have shown to be successful for imaging with promising tumor-to-background ratios on PET/CT scans in various solid tumors (Kratochwil et al. 2019); however, their limited tumor retention time makes these molecules suboptimal for radionuclide therapy. We aimed to develop a novel FAP-targeting pharmacophore with improved pharmacokinetics, which could function as a core structure for FAP-targeting oncological interventions. The DOTA-conjugated eFAP-6 and sCy5-conjugated eFAP-7, based on the 8-QCP core structure, were successfully synthesized using a multistep procedure. Both eFAP compounds demonstrated remarkable inhibitory potency against human and murine FAP isoforms, as well as good selectivity. This confirms that introducing a chelator or another payload at the 8-position can be performed while maintaining outstanding FAP affinity. [^111^In]In-eFAP-6 and [^177^Lu]Lu-eFAP-6 were obtained in high radiochemical yield and purity, and demonstrated high stability in PBS, MS, and HS, for up to 24 h. [^111^In]In-eFAP-6 demonstrated a highly hydrophilic character; however, the payload change from a DOTA-chelator to sCy5 caused eFAP-7 to exhibit higher lipophilic properties, inherent with the sCy5 lipophilicity. In addition, some loss of stability in HS and MS after 24 h incubation was observed. The sCy5 dye is a bulky chemical moiety compared to the DOTA-chelator, but it should have less impact on the pharmacokinetics and hydrophilicity of the small molecule than alternative larger dyes (e.g., IRDye800CW) (Zettlitz et al. 2019). To improve long-term stability, conjugation with other dyes can be evaluated, although the selection of the optimal dye also depends on the application of the compound (e.g., preclinical fluorescent imaging or FGS).

The affinity of eFAP-6 and eFAP-7 was further confirmed in huFAP-expressing cells and tumor xenografts. Cell uptake studies demonstrated a high FAP-mediated uptake of [^111^In]In-eFAP-6, only slightly lower than that of [^111^In]In-FAPI-46. However, a distinct uptake pattern for [^111^In]In-eFAP-6 was observed, unlike that of [^111^In]In-FAPI-46 or reported for other FAP-targeting compounds. A decline in uptake over time has been reported for other FAP-targeting small molecules (Li et al. 2022; Yang et al. 2023), but other studies did not report on the radiotracer uptake within two hours of incubation, making it difficult to establish if other FAP-targeting small molecules exhibit the same pattern. Prior research into FAP trafficking to the cell surface indicated that FAP dimerization occurs intracellularly (Wonganu and Berger 2016). Possibly eFAP-6 has an alternative effect on FAP returning to the membrane after internalization, by affecting this dimerization and trafficking to the cell membrane and limiting re-uptake of [^111^In]In-eFAP-6. Another theory is that the binding of eFAP-6 to FAP could result in FAP shedding from the membrane; however, little is known about FAP shedding in vitro (Lindner et al. 2019; Lee et al. 2006). More research is needed to understand the mechanisms of FAP dimerization, cell membrane availability, and how this affects tracer processing after binding. Such future studies should ideally be performed in non-transduced FAP-expressing human cancer cell lines or CAFs, since this likely reflects the physiological FAP expression and its interaction with FAP-targeting radiotracers more accurately compared to cancer cell lines transduced to express human FAP.

The difference in peak uptake between eFAP-6 and FAPI-46 was less prominent in our in vivo studies with the HT1080-huFAP xenografted mice. The data demonstrated that [^177^Lu]Lu-eFAP-6 had rapid uptake that peaked early on, but at 24 h p.i. less uptake remained in the tumor xenografts than for [^177^Lu]Lu-FAPI-46, indicating that enhanced tumor retention was not accomplished with eFAP-6. Currently, an improved in vivo tumor retention up to three days has been shown with the cyclic peptide FAP-2286, resulting in improved FAP-TRT efficacy (Zboralski et al. 2022). Another strategy to improve tumor retention that is being explored, is the development of homodimers of FAP-targeting agents. For example, BiOncoFAP and DOTA.(SA.FAPi)_2_ have both demonstrated improved tumor residence time (Galbiati et al. 2022; Moon et al. 2021), partly due to a longer circulation time. However, there is still room for optimization of the small molecule monomers to provide rapid clearance from the blood and non-target organs, while improving tumor retention.

Although eFAP-6 did not show improved tumor retention, it did demonstrate rapid clearance from non-target organs and beneficial tumor-to-background ratios at 1 and 4 h p.i. These pharmacokinetic properties make eFAP-6 suitable for nuclear imaging. Especially when labeled with the short-lived positron emitter gallium-68, eFAP-6 could be a promising candidate for PET/CT scans. In addition, the rapid washout of non-target organs indicates that there is low off-target toxicity, which is beneficial when applying targeted α-therapy with a short lived isotope. Bismuth-213 would be a promising candidate with a half-life of 45.6 min and a high linear energy transfer, matching the short biological half-life of eFAP-6 (Ahenkorah et al. 2021). Preclinical studies applying TRT with bismuth-213 directed against PSMA in prostate cancer cells have shown promising results (Ballangrud et al. 2001; Nonnekens et al. 2017; McDevitt et al. 2000). Consequently, the development of labeling protocols for [^68^Ga]Ga-eFAP-6 and [^213^Bi]Bi-eFAP-6 would be needed, as well as stability tests, to explore the safety and efficacy of this theranostic pair. However, optimization of eFAP-6 would first be necessary to alter its excretion pathway, with the aim of decreasing the liver uptake and promoting renal clearance.

Similar to eFAP-6, the fluorescent tracer eFAP-7 also demonstrated rapid and specific uptake in both HT1080-huFAP and PS-1 cells. Especially in the high FAP-expressing HT1080-huFAP cells, the uptake was already visible at 15 min and remained at 45 min of incubation. This compound could be used for in vitro imaging to study the interactions between FAP and FAP-targeting tracers. In line herewith, the confocal microscopy images indicated that eFAP-7 was rapidly internalized in the cell and collected in the perinuclear region. The later could indicate that the tracer was transported towards the endoplasmic reticulum in lysosomes for degradation (Bouley et al. 2005). Future research, including quantitative measurements and live cell imaging, combined with lysosomal trackers over time, is necessary to confirm this degradation pathway. Moreover, such fluorescent molecules can be used to understand how chemical alterations in the structure of the molecules impacts their uptake and retention time.

The sCy5-conjugated eFAP-7 could also be used for preclinical in vivo imaging or even FGS (Verhoeven et al. 2023; Pagoto et al. 2020). Regarding the latter, due to the currently available equipment and experience in the clinic, an IRDye800CW might be preferred (Rosenthal et al. 2015). Nevertheless, as the sCy5 is less bulky, it could be more suitable for a small molecule like ours. Besides, sCy5 has an improved solubility over an IRDye800CW (Zettlitz et al. 2019). Thus, eFAP-7 should be evaluated in in vivo studies to determine its biodistribution and pharmacokinetics. This compound could have great potential in FGS for many solid cancers, to aid in resecting the full tumor, including its malignant stroma.

## Conclusion

Here we describe a novel FAP-targeting pharmacophore that was used to synthesize the radiotracer eFAP-6 and fluorescent tracer eFAP-7. The in vitro and in vivo analyses of these compounds have given us insight into the effect of a substitution at the 8-position of the quinoline ring, which is a first step in developing a FAP-targeting core structure with (radio)theranostic potential. In future studies, we aim to further alter this 8-QCP core structure to improve retention with the ultimate goal of developing an effective FAP-directed TRT strategy. In addition, optimizing the eFAP pharmacophore will be the basis for other FAP-targeting oncological interventions, aiming to develop better probes for FGS, multimers, and drug conjugates.

## Methods

### Chemistry and radiochemistry

General information on the synthetic methods allowing preparation of eFAP-6 and eFAP-7 are described in the supplementary data.

Both eFAP-6 and eFAP-7 were synthesized through a multi-step organic synthesis procedure. Detailed information on the synthesis and characterizations is provided in the Supplementary Materials (Figures S1-S9). eFAP-6 was titrated prior to the labeling, following previously described methods (Breeman et al. 2014). Labeling reactions were performed in a solution containing sodium acetate (1 µL, 2.5 M), ascorbic and gentisic acids (10 µL, 50 mM), ethanol (10 µL), and complemented with Milli-Q water. eFAP-6 (1 nmol), 50 MBq [^111^In]InCl_3_ (Curium Pharma, Petten, The Netherlands) or 13–20 MBq [^177^Lu]LuCl_3_ (IDB Group, Baarle-Nassau, The Netherlands) were then added, and the reaction mixture was heated at 90 °C for 15 min. [^111^In]In-eFAP-6 at a molar activity of 50 MBq/1 nmol and [^177^Lu]Lu-eFAP-6 at 20 MBq/1 nmol were used for in vitro studies, [^177^Lu]Lu-eFAP-6 was prepared at 13 MBq/1 nmol for the in vivo studies. The incorporation of radioactivity was assessed using iTLC-SG plates (Agilent; Folsom, CA, USA) eluted with a solution of sodium citrate (0.1 M, pH 5.0), analyzed by a bSCAN scanner (Brightspec; Antwerp, Belgium) and expressed as the radiochemical yield. The radiochemical purity was determined using HPLC. Diethylenetriaminepentaacetic acid (DTPA) (5 µL, 4 mM) was added to complex-free indium-111 or lutetium-177 before injection onto analytical radio-HPLC.

### Stability studies in PBS, human, and mouse serum

For the stability assessment of eFAP-6, [^111^In]In-eFAP-6 or [^177^Lu]Lu-eFAP-6 (~ 3–7 MBq, 20 µL) was mixed with 180 µL of PBS (0.01 M, pH 7.4) and incubated at 37 °C for up to 24 h. A sample of the PBS solution was directly injected on the radio-HPLC at 1, 4, and 24 h after incubation. Additionally, [^111^In]In-eFAP-6 and [^177^Lu]Lu-eFAP-6 (5–15 MBq, 50 µL) were incubated with 350 µL of commercially acquired mouse or human serum (Biolegend; San Diego, CA, USA) at 37 °C for up to 24 h. Following 1, 4, and 24 h of incubation, 50 µL of the solution were mixed with 50 µL of acetonitrile in an Eppendorf tube, and centrifuged at 5000× *g* for 20 min. The supernatant was then analyzed by radio-HPLC. Stability studies of eFAP-7 were performed by incubation of eFAP-7 (50 µL, 10^− 4^ M) in 450 µL PBS or serum at 37 °C for up to 24 h. After 1, 4, and 24 h of incubation, 50 µL of the PBS incubation solution were directly analyzed by liquid chromatography-mass spectrometry (LC-MS), while 50 µL of the serum solution was mixed with 50 µL of acetonitrile in an Eppendorf tube, and centrifuged at 5000× g for 20 min, followed by LC-MS analyses.

### Determination of LogD_7.4_ value

The distribution coefficients (LogD_7.4_) were determined using a shake-flask method, in which 2 µL (~ 0.5 MBq) of [^111^In]In-eFAP-6 or 10 µL of eFAP-7 (10^− 4^ M) was added to an Eppendorf tube containing 1 mL of PBS (0.01 M, pH 7.4) and n-octanol (*v*/*v*, 1:1). After vigorous vortexing, the solution was centrifuged at 2500× *g* for 15 min for phase separation. For radiolabeled eFAP-6, samples (3 × 10 µL) of the octanol and aqueous phases were taken out and measured in a 1480 Wizard automatic γ-counter (PerkinElmer; Groningen, The Netherlands). LogD_7.4_ values were calculated by using the following equation: LogD_7.4_ = log [(counts in octanol phase)/(counts in aqueous phase)]. For eFAP-7, samples (50 µL) of the octanol and aqueous phases were taken out and measured by LC-MS. LogD_7.4_ values were calculated by using the following equation: LogD_7.4_ = log [(area in octanol phase)/(area in aqueous phase)].

### In vitro inhibition studies on huFAP, muFAP, PREP and DPP4

All enzymatic assays were performed in black 384-well plates, and enzymatic activity was measured on a microplate reader, monitoring the fluorescence at λ_EX_ 350 nm/λ_EM_ 485 nm. For huFAP and muFAP, 50 µL of reaction mixture consisting of the substrate (Z-Gly-Pro-AMC, 10 µM), huFAP or muFAP protein (10 ng), assay buffer (50 mM Tris, 100 mM NaCl, and 1.5 µM BSA, DMSO (1% v/v), pH 7.4), were supplemented with increasing concentrations of inhibitors eFAP-6, eFAP-7, or FAPI-46 (10^− 11^ M to 10^− 5^ M). Val-boroPro (10^− 4^ M) and assay buffer served as a positive and negative control, respectively. To measure inhibition of PREP, 50 µL of the reaction mixture containing 1 µM Z-Gly-Pro-AMC and 10 ng PREP (R&D Systems; Minneapolis, MN, USA) were used. The enzymatic assay of DPP4 contained substrate (100 µM), DPP4 (Abcam; Cambridge, UK) (10 nM), assay buffer, and increasing concentrations of the inhibitors (10^− 10^ M to 10^− 4^ M). Sitagliptin (10^− 4^ M) and assay buffer were used as a positive and negative control, respectively. H-Gly-Pro-AMC (100 µM) in assay buffer was used as a baseline control. The plates were analyzed in a Hidex Sense Microplate reader (Hidex; Turku, Finland) at 37 °C under dark conditions for 1 h (huFAP), 2 h (muFAP), or 30 min (PREP, DPP4). For all assays, the components were kept on ice before plating.

### Cell culture

Cell experiments were performed with the human sarcoma HT1080 cell line, stably transduced with human FAP (HT1080-huFAP) which were kindly provided by Prof. Dr. Uwe Haberkorn from the University of Heidelberg. The wild type HT1080 (ATTC), hereafter HT080-WT, was used as a negative control. Additionally, the pancreatic stellate cells PS-1, obtained from Queen Mary University, were used as an endogenously FAP expressing cell line. HT1080-huFAP and HT1080-WT cells were cultured in DMEM Glutamax© (Gibco; Breda, The Netherlands) and the PS-1 cells were cultured in RPMI (Gibco). Both media were supplemented with 10% FBS (Gibco), 100 UI/mL penicillin, and 100 µg/mL streptomycin (Merck Life Science NV; Amsterdam, The Netherlands). All cell lines were regularly passaged at 80% confluence and cultured at 37 °C, 5% CO_2_.

### eFAP-6 and eFAP-7 displacement and uptake assays

In vitro displacement studies of eFAP-6 and eFAP-7, as well as specificity and uptake assays, were performed using HT0180-huFAP cells. One day prior to the assay 100,000 cells were seeded in a 12-well plate in triplicate. For the displacement assay, cells were incubated for 45 min with [^111^In]In-FAPI-46 simultaneously with increasing concentrations of unlabeled eFAP-6, eFAP-7, or FAPI-46 (10^− 12^ to 10^− 6^ M). For the uptake assay, the cells were incubated with 1 nM [^111^In]In-eFAP-6 or [^111^In]In-FAPI-46 for different time points (5–120 min) in the presence and absence of 1 mM UAMC-1110 to determine the specificity of^111^ radiotracer binding. In both assays, the cells were washed twice with PBS after incubation and were subsequently lysed using 1 M NaOH for 20 min. The cell lysates were collected and radioactivity was measured in a γ-counter (PerkinElmer), simultaneously with 100 µL of the radioactive incubation medium as a standard. Cells in separate wells were collected with Trypsin-EDTA (Gibco) for counting by the automated Countess II cell counter (Thermo Fisher Scientific; Bleiswijk, The Netherlands). Uptake was expressed as percentage added activity per 200,000 cells (% AA/200,000 cells), and specific uptake was corrected by subtracting the uptake measured in the block wells.

### Confocal microscopy

Fluorescent tracer uptake was analyzed on HT1080-huFAP, HT1080-WT, and PS-1 cells. One day prior to the assay 280,000 HT1080-huFAP, 300,000 HT1080-WT, and 460,000 PS-1 cells were seeded on 100 mm coverslips in a 6-well plate. The next day cells were incubated with 1 nM of eFAP-7 in the presence and absence of 1 mM UAMC-1110 to determine the specificity of the uptake. Cells incubated only in medium were taken along to correct for auto-fluorescence. After 15 and 45 min of incubation, the cells were washed twice with cold PBS and fixed by incubation in 1 mL of cold 2% paraformaldehyde for 15 min at 4 °C. Subsequently, the coverslips were mounted with Vectashield^®^ Antifade Mounting Medium with DAPI (Vector Laboratories; Newark, CA, USA) and imaged with a Leica SP8 confocal microscope using a 40x objective (Leica Microsystems; Wetzlar, Germany). Data were acquired with the same microscope settings for all acquisitions, with the parameters λ_EX_ 405 nm/λ_EM_ 420–470 nm for DAPI and λ_EX_ 633 nm/λ_EM_ 666 nm for sCy5. Analyses were performed in Fiji (Schindelin et al. 2012) and were subjected to a custom-made macro to correct for auto-fluorescence signal. The images were linear contrast stretched with DAPI contrast borders at 0-150 for cell lines. The contrast borders for the sCy5 were 15–155 for HT1080-huFAP, 14–100 for PS-1, and 30–255 for HT1080-WT, respectively.

### Biodistribution study

All animal experiments were approved by the Erasmus MC Animal Welfare Committee and were in accordance with European law. Five-week-old male NMRI-Foxn1 nu/nu mice (Janvier; Le Genest-Saint-Isle, France) were housed in individually ventilated cages with a maximum of three mice per cage. After one week of acclimatization, mice were subcutaneously inoculated with HT1080-huFAP on the right shoulder and HT1080-WT on the left shoulder (2.5 × 10^6^ cells in Hank’s Balanced Salt Solution (Gibco)). Tumors were allowed to grow for 10 days, resulting in tumor sizes of 537 ± 269 mm^3^ and 305 ± 190 mm^3^ for HT1080-huFAP and HT1080-WT, respectively, at the start of the experiment. The animals received 200 µL of [^177^Lu]Lu-eFAP-6 (n=3/4 per group) or [^177^Lu]Lu-FAPI-46 (n=3/4 per group) (2.6 MBq/200 pmol) by intravenous injection through the tail vein, andat 1, 4, and 24 h p.i. biodistribution studies were performed. Blood was collected by orbital puncture under isoflurane/O_2_ anesthesia, immediately followed by cervical dislocation. Hereafter the two tumor xenografts, pancreas, liver, GI-tract (stomach, small intestine, cecum, colon), kidneys (left and right), lungs, heart, salivary glands, muscle, and bone (femur), were collected, weighed, and measured in a y-counter (PerkinElmer), alongside 10 µL of the injected activity as standard. The results are expressed as the percentage injected dose per gram (% ID/g).

### Statistics

All statistical analyses were performed with GraphPad Prism version 9.0 (San Diego, California USA). All IC_50_ values were determined using a non-linear regression Log(inhibitor) vs. response analysis with variable slope (four parameters). For the in vivo results outliers were identified by a ROUT test (Q = 1%) and excluded. Next, a two-tailed student’s t-test was performed (*p < 0.05, **p < 0.01, ***p < 0.001). All experiments were performed in triplicate and repeated at least three times.

### Electronic supplementary material

Below is the link to the electronic supplementary material.

Supplementary Material 1

## Data Availability

All available data are described in the manuscript and available through the supplementary material. Additional information can be obtained by contacting the authors upon reasonable request. The following supporting data and materials can be found in the supplementary materials: Figure S1: ^1^H NMR of **1** in chloroform-d, Figure S2: ^13^C NMR of **1** in chloroform-d, Figure S3: LC-MS spectrum of **1**, Figure S4: ^1^H NMR of eFAP-6 in D_2_O, Figure S5: ^13^C NMR of eFAP-6 in D_2_O, Figure S6: LC-MS spectrum of eFAP-6, Figure S7: LC-MS spectrum of eFAP-7, Figure S8: Radio-HPLC chromatograms of [^111^In]In-eFAP-6 in PBS, Figure S9: Radio-HPLC chromatograms of [^111^In]In-eFAP-6 in mouse serum, Figure S10: Radio-HPLC chromatograms of [^111^In]In-eFAP-6 in human serum, Figure S11: Radio-HPLC chromatograms of [^177^Lu]Lu-eFAP-6 in PBS, Figure S12: Radio-HPLC chromatograms of [^177^Lu]Lu-eFAP-6 in mouse serum, Figure S13: Radio-HPLC chromatograms of [^177^Lu]Lu-eFAP-6 in human serum, Figure S14: LC chromatograms of eFAP-7 in PBS, Figure S15 LC chromatograms of eFAP-7 in mouse serum, Figure S16: LC chromatograms of eFAP-7 in human serum, Figure S17: In vitro uptake and blocked uptake experiment on HT1080-huFAP, Figure S18: Confocal microscopy HT1080-WT cells, and Table 1 Ex vivo biodistribution of [^177^Lu]Lu-FAPI-46 and [^177^Lu]Lu-eFAP-6.

## Abbreviations

% AA: Percentage added activity
Boc: Tert-butyloxycarbonyl
CAF: Cancer-associated fibroblast
DPP4: Dipeptidyl peptidase IV
DTPA: Diethylenetriaminepentaacetic acid
FAP: Fibroblast activation protein
FGS: Fluorescent-guided surgery
GI-tract: Gastro-intestinal tract
h: Hour
HPLC: High-performance liquid chromatography
HS: Human serum
huFAP: Human FAP
LC-MS: Liquid chromatography-mass spectrometry
min: Minutes
MS: Mouse serum
muFAP: Murine FAP
na: Not available
ns: Non-significant
PBS: Phosphate-buffered saline
p.i.: Post injection
PREP: Prolyl oligopeptidase
QCP: (4-quinolinoyl)-glycyl-2-cyanopyrrolidine
sCy5: Sulfo-Cyanine5
SI: Selectivity index
TBR: Tumor-to-background ratio
TME: Tumor microenvironment
TRT: Targeted radionuclide therapy
WT: Wild type
