# Supplementary material for: In vitro and in vivo analyses of eFAP: a novel FAP-targeting small molecule for radionuclide theranostics and other oncological interventions
Source: EJNMMI Radiopharm Chem. 2024 Jul 29;9:55. doi: 10.1186/s41181-024-00283-x (PMC11286609; doi:10.1186/s41181-024-00283-x)
Supplement: Supplementary file 1 — Supplementary Material 1 [file 41181_2024_283_MOESM1_ESM.docx]

**Supplementary Information:**

**In vitro and in vivo analyses of eFAP: a novel FAP-targeting small molecule for radionuclide theranostics and other oncological interventions**

*Circe D. van der Heide, Hanyue Ma, Mark W.H. Hoorens, Joana D. Campeiro, Debra C. Stuurman, Corrina M.A. de Ridder, Yann Seimbille, and Simone U. Dalm.*

***Supplementary materials and methods***

*S1 General information materials and methods*

Instant thin-layer iTLC-SG chromatography plates were analyzed by a bSCAN scanner (Brightspec; Antwerp, Belgium). Radioactivity in biological and logD_7.4_ experiments was measured using a PerkinElmer Wizard 2 gamma counter (Groningen, The Netherlands). Radio-HPLC was performed with a Waters Alliance e2695 system (Etten-Leur, The Netherlands) equipped with a 2998 diode array (PDA) detector for UV detection; a NaI(Tl) Scionix crystal (Bunnik, The Netherlands) connected to a Canberra Osprey multichannel analyzer and a signal amplifier (Zellik, Belgium) for detection of the radioactive signal. The reversed-phase analytical Gemini C18 column (250 × 4.6 mm, 5 µm) from Phenomenex (Torrance, CA, USA) was used for all analyses. The mobile phase consisted of the following; solvent A, 0.1% trifluoroacetic acid (TFA) in H_2_O; Solvent B, 0.1% TFA in acetonitrile (ACN). Analysis was performed at a flow rate of 1 mL/min using the following gradient of solvents A and B: 0–3 min, 5% B; 3–23 min, 5–100% B; 23–27 min, 100% B. high-performance liquid chromatography (HPLC) eluates were monitored for their UV absorbance at 254 nm. Corning® Molecular Biology Grade Water (Corning; Amsterdam, The Netherlands) was referred to as H_2_O. All recombinant protein assays were performed in 384-well plates (nonbinding, ps, f-bottom, black, high volume).

*S2 General information on synthetic methods*

All chemicals were obtained from commercial suppliers in reagent grade or better and were used without further purification unless specified. All solvents were of analytical or HPLC grade. Reactions were magnetically stirred and monitored by thin-layer chromatography (TLC) on silica gel 60 F254 aluminum-backed pre-coated plates (Merck; Amsterdam, The Netherlands). Preparative HPLC was carried out using an Agilent 1290 Infinity II Preparative LC System (Middelburg, The Netherlands) equipped with a 1260 Infinity II autosampler, 1260 Infinity II fraction collector, and Agilent 5 Prep C18 column (50 x 21.2 mm), measuring UV absorbance at 230, 254 and 280 nm. The mobile phase consisted of the following: Solvent A, 0.1% formic acid (FA) in water; Solvent B, 0.1% FA in ACN. The default gradient used was 0–8 min, 5–100% B, and 8–10 min, 100% B. The flow rate was 10 mL/min, and chromatograms were recorded using the Agilent OpenLab CDS Chemstation software. Liquid chromatography-mass spectrometry (LC-MS) was carried out on an Agilent 1260 Infinity II electrospray ionization (ESI) LC-MS system equipped with an Agilent InfinityLab Poroshell 120 EC-C18 column (3.0 x 100 mm, 2.7 μm). Products were eluted using a gradient elution of acetonitrile from 5% to 100% in H_2_O, containing 0.1% FA at a flow rate of 0.5 mL/min for 5 min, and monitored at 220, 254, and 280 nm by a UV detector. All final compounds showed a single peak at the designated retention time and were at least 95% pure. Melting points were determined using a Stuart SMP20. NMR spectra were recorded in DMSO-d_6_, D_2_O, CDCl_3_, or CD_3_OD in diluted solutions on a 60 MHz Nanalysis NMReady 60Pro (Calgary, Canada) at ambient temperature. Chemical shifts are given as *δ* values in ppm and coupling constants *J* are given in Hz. The splitting patterns are reported as s (singlet), d (doublet), t (triplet), q (quartet), m (multiplet), dd (doublet of doublets), and br (broad signal). Lyophilization was achieved under reduced pressure using a FreeZone Benchtop Freeze Dryer (Labconco; Kansas City, MO, USA).

*S3 Synthesis of eFAP-6 and eFAP-7*

***Synthesis of tert-butyl (S)-(3-((4-((2-(2-cyano-4,4-difluoropyrrolidin-1-yl)-2-oxoethyl)carbamoyl)quinolin-8-yl)oxy)propyl)carbamate (1)***
(1) was carried out according to a synthetic method adapted from the protocols previously reported by Lindner and coworkers [24]. ^1^H NMR (60 MHz, chloroform-*d*) *δ* 8.69 (d, *J* = 4.2 Hz, 1H), 7.64 (m, 2H), 7.45 – 7.02 (m, 2H), 6.86 (d, *J* = 7.3 Hz, 1H), 6.30 (s, 1H), 4.82 (s, 1H), 4.02 (m, 4H), 3.22 (m, 2H), 2.55 (m, 4H), 2.04 (m, 2H), 1.35 (s, 9H). ^13^C NMR (15 MHz, chloroform-*d*) *δ* 167.79, 156.37, 154.36, 148.47, 141.01, 140.39, 127.84, 125.35, 119.42, 117.00, 116.49, 109.72, 78.65, 68.24, 49.86, 44.43, 42.02, 38.81, 38.38, 29.19, 28.39. (CF_2_ not detected). ESI-MS *m/z*: calculated for C_25_H_29_F_2_N_5_O: 517.21; found: 517.3 [M+H]^+^.

***Synthesis of (S)-2,2',2''-(10-(2-((3-((4-((2-(2-cyano-4,4-difluoropyrrolidin-1-yl)-2-oxoethyl)carbamoyl)quinolin-8-yl)oxy)propyl)amino)-2-oxoethyl)-1,4,7,10-tetraazacyclododecane-1,4,7-triyl)triacetic acid (eFAP-6)***.

**1** (0.02 g, 0.04 mmol) was added a 1:1 mixture of TFA/DCM containing 10% TIPS (1.5 mL), after which the reaction mixture was left to stir at room temperature (RT) for 30 min. Following completion, the reaction mixture was concentrated under a gentle air stream, before precipitating with ice cold diethyl ether (2 x 20 mL). The precipitate **2** was collected, dried, and used in the subsequent reaction without further purification. The crude was redissolved in DMF (1 mL), after which DOTA-NHS ester (0.035 g, 0.043 mmol) and DIPEA (45 µL) were added. The reaction mixture was stirred at RT for 30 min. The volatiles were removed under reduced pressure. The product was purified using preparative high-performance liquid chromatography followed by lyophilization to yield eFAP-6 (12.3 mg, 0.015 mmol, 38%) as a light-yellow powder. ^1^H NMR (60 MHz, D_2_O) δ 9.03 (dd, *J* = 4.9, 1.6 Hz, 1H), 8.41 (d, *J* = 1.5 Hz, 0H), 7.94 (dd, *J* = 5.1, 1.5 Hz, 1H), 7.89 – 7.84 (m, 1H), 7.80 (td, *J* = 8.2, 1.6 Hz, 1H), 7.49 (dd, *J* = 8.0, 1.3 Hz, 1H), 5.21 (dd, *J* = 9.0, 3.8 Hz, 1H), 4.64 – 4.51 (m, 0H), 4.46 – 4.38 (m, 4H), 4.31 (td, *J* = 13.0, 6.6 Hz, 1H), 4.25 – 4.14 (m, 1H), 4.08 (dd, *J* = 17.1, 11.9 Hz, 0H), 3.84 (d, *J* = 16.6 Hz, 2H), 3.76 (d, *J* = 16.1 Hz, 2H), 3.55 – 3.32 (m, 12H), 3.19 – 2.84 (m, 9H), 2.22 (p, *J* = 6.4 Hz, 2H). ^13^C NMR (151 MHz, D_2_O) δ 175.65, 171.90, 169.81, 169.67, 169.23, 169.07, 151.76, 146.49, 144.89, 135.38, 130.10, 127.51, 125.73, 120.09, 117.34, 116.63, 112.01, 67.17, 56.33, 55.83, 54.17, 51.96, 51.74, 51.58, 50.68, 48.28, 48.12, 44.76, 42.11, 36.67, 36.40, 36.23, 27.69. ESI-MS *m/z*: calculated for C_36_H_47_F_2_N_9_O_10_: 803.34; found: 804.3 [M+H]^+^.

***Synthesis of 1-(6-((3-((4-((2-((S)-2-cyano-4,4-difluoropyrrolidin-1-yl)-2-oxoethyl)carbamoyl)quinolin-8-yl)oxy)propyl)amino)-6-oxohexyl)-3,3-dimethyl-5-sulfo-2-((1E,3E)-5-((E)-1,3,3-trimethyl-5-sulfoindolin-2-ylidene)penta-1,3-dien-1-yl)-3H-indol-1-ium (eFAP-7)***.

**1** (5.0 mg, 9.7 µmol) was added a 1:1 mixture of TFA/DCM containing 10% TIPS (1 mL), after which the reaction mixture was left to stir at RT for 30 min. Following completion, the reaction mixture was concentrated under a gentle air stream, before precipitating with ice cold diethyl ether (2 x 20 mL). The precipitate **2** was collected, dried, and used in the subsequent reaction without further purification. The residue was redissolved in DMF (1 mL) and sulfo-Cy5-NHS ester (6.7 mg, 8.7 µmol) and DIPEA (10 µL) were added. The reaction mixture was stirred at RT for 45 min. The volatiles were removed under reduced pressure. The crude was purified using preparative high-performance liquid chromatography followed by lyophilization to yield eFAP-7 (5.2 mg, 5.0 µmol, 52%) as a bright blue power. ESI-MS m/z: calculated for C_52_H_58_F_2_N_7_O_10_S_2_^+^ 1042.35; found. 1042.6 [M + H]^+^.

***Supplementary figures and tables***


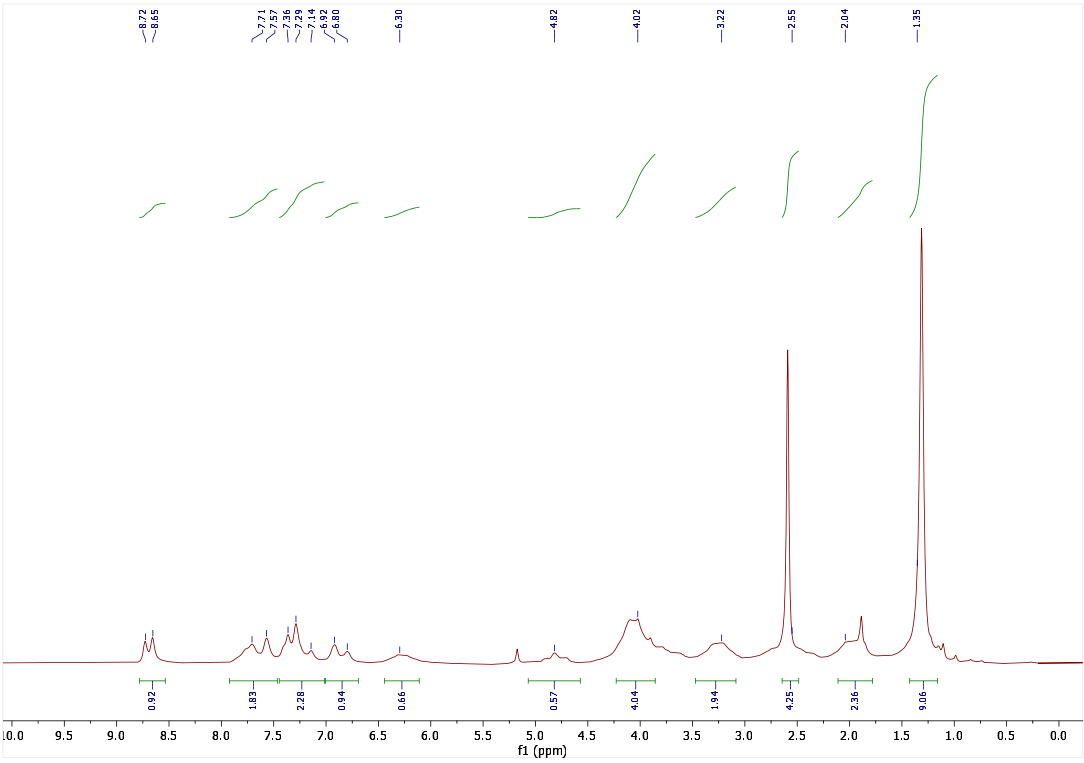


**Figure S1:** ^1^H NMR of **1** in chloroform-d.


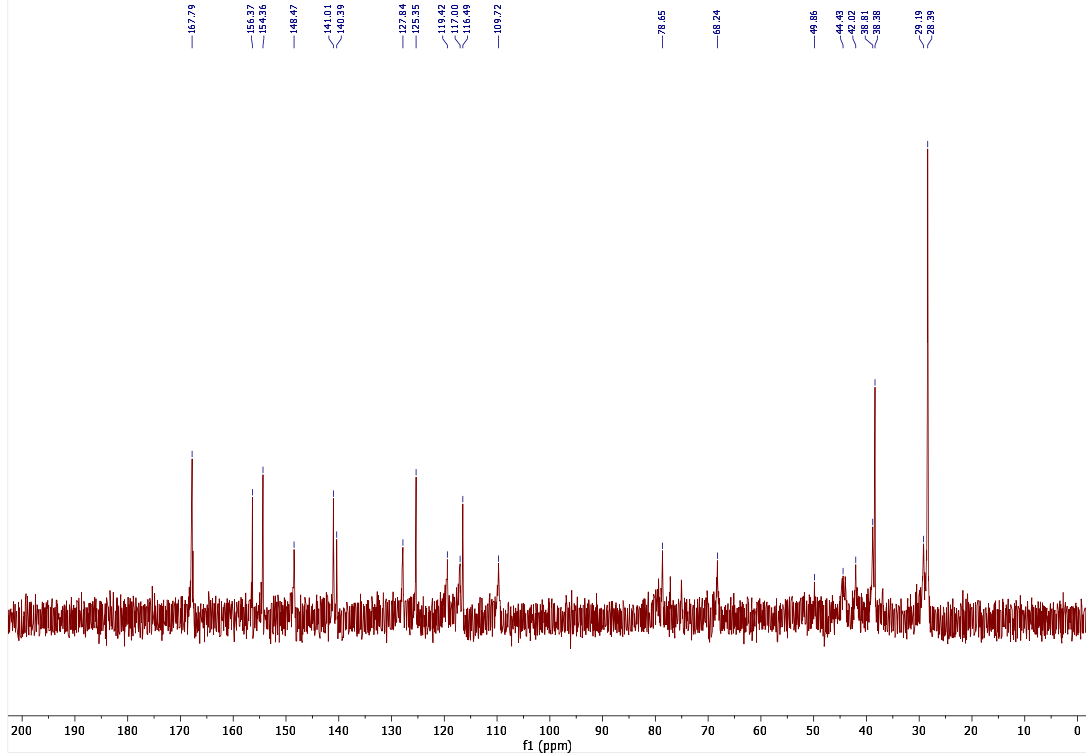


**Figure S2:** ^13^C NMR of **1** in chloroform-d.


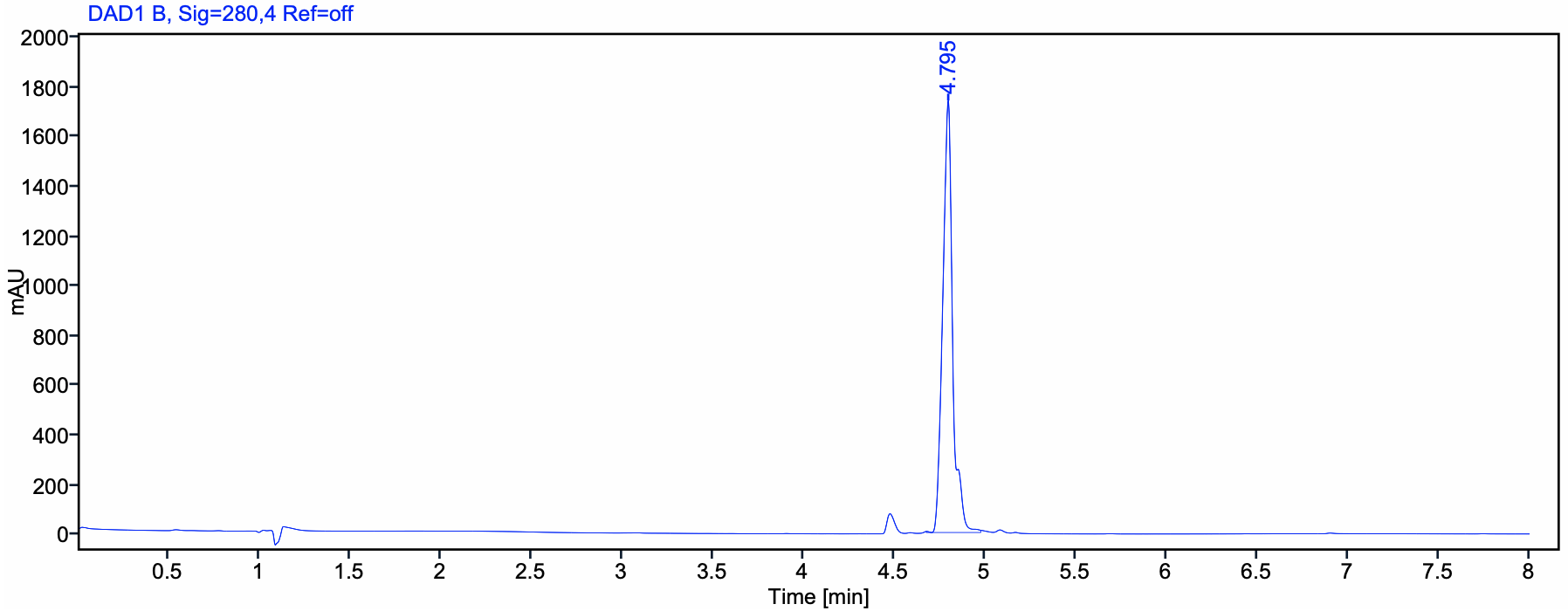

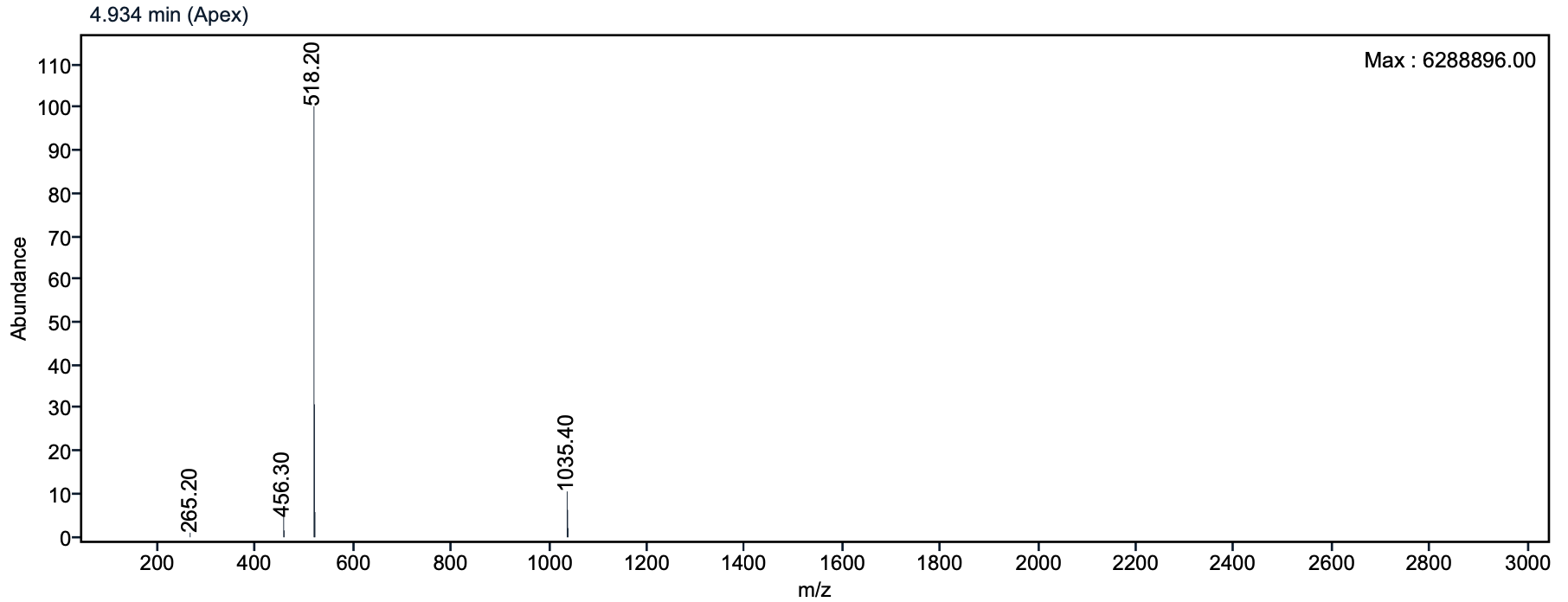


**Figure S3:** LC-MS spectrum of **1.**

**Figure S4**: ^1^H NMR of eFAP-6 in D_2_O.

**Figure S5**: ^13^C NMR of eFAP-6 in D_2_O.


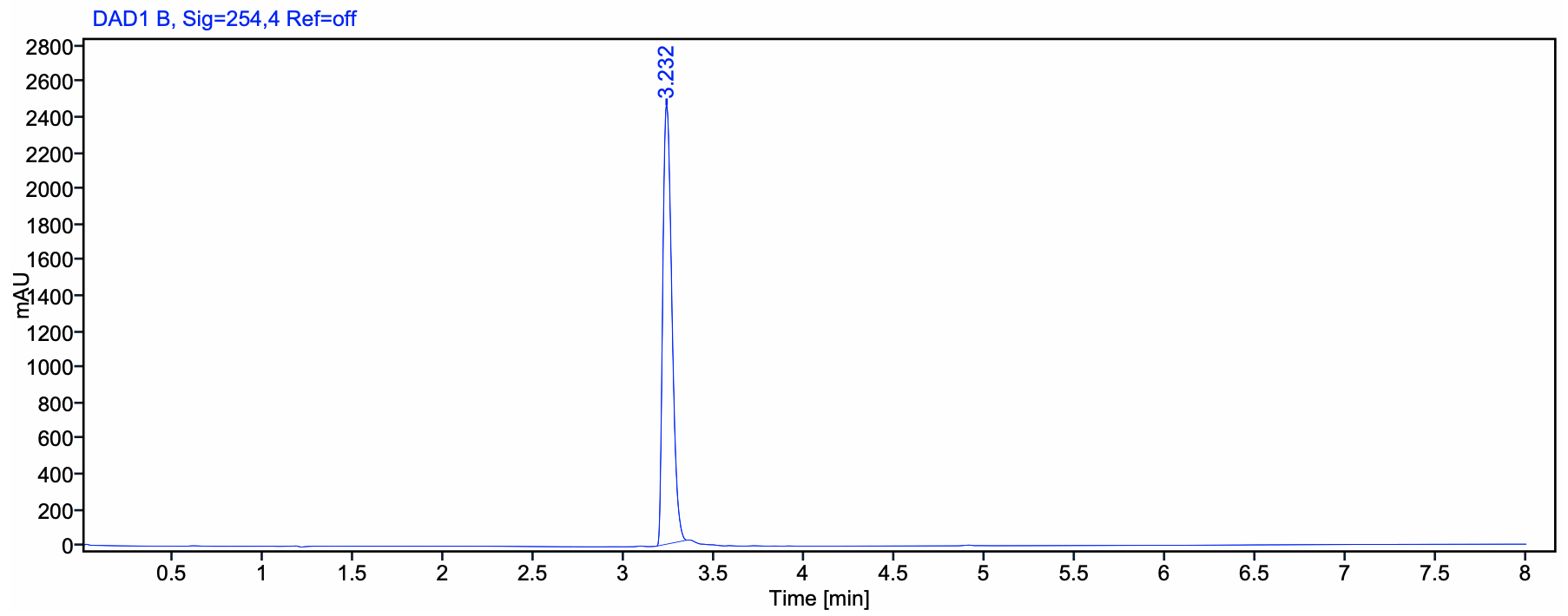

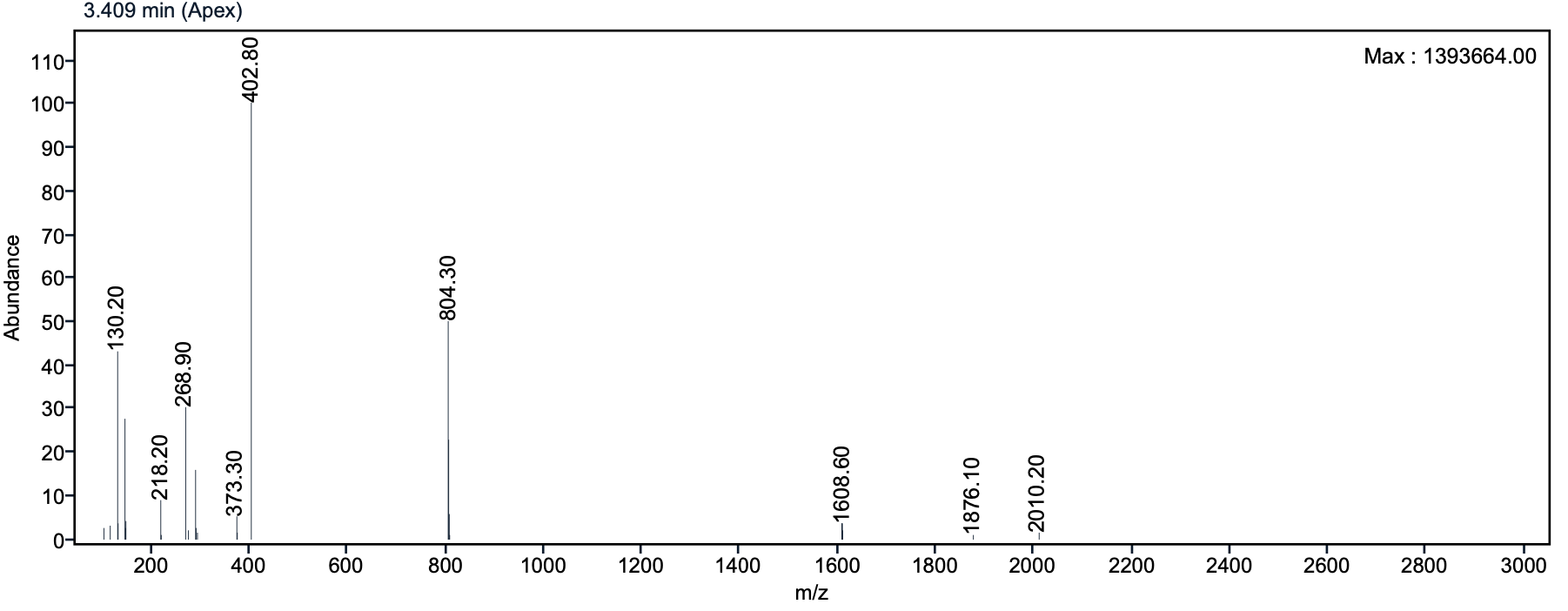


**Figure S6:** LC-MS spectrum of eFAP-6**.**

**
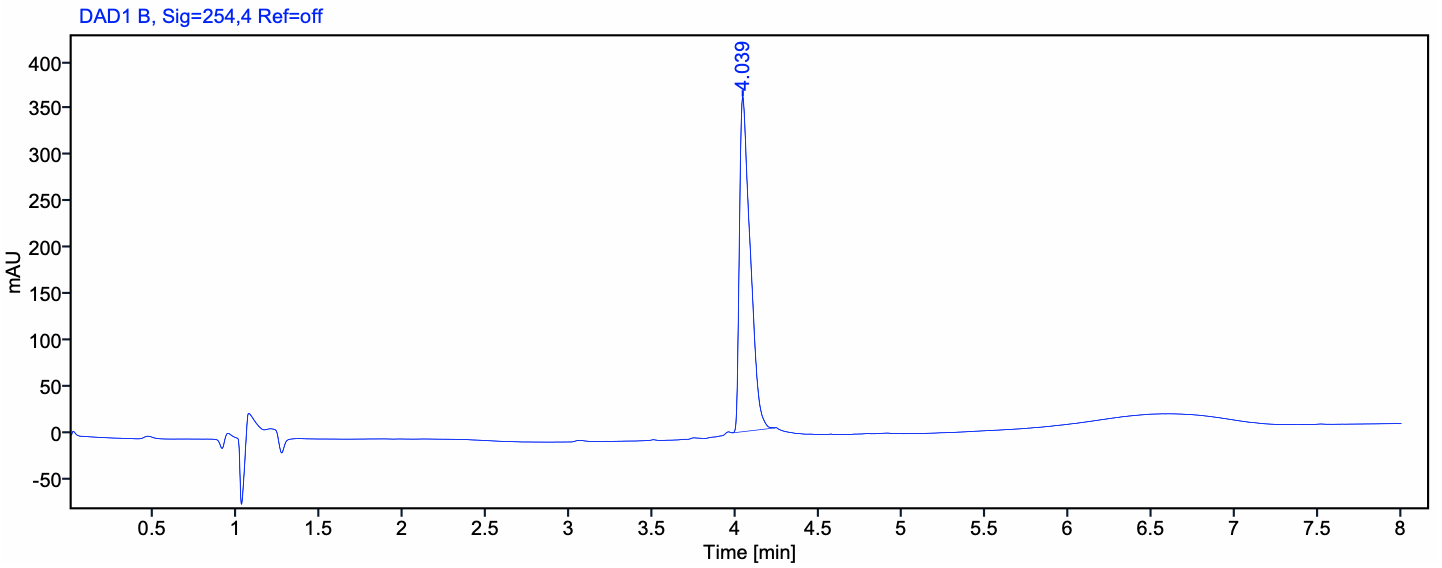

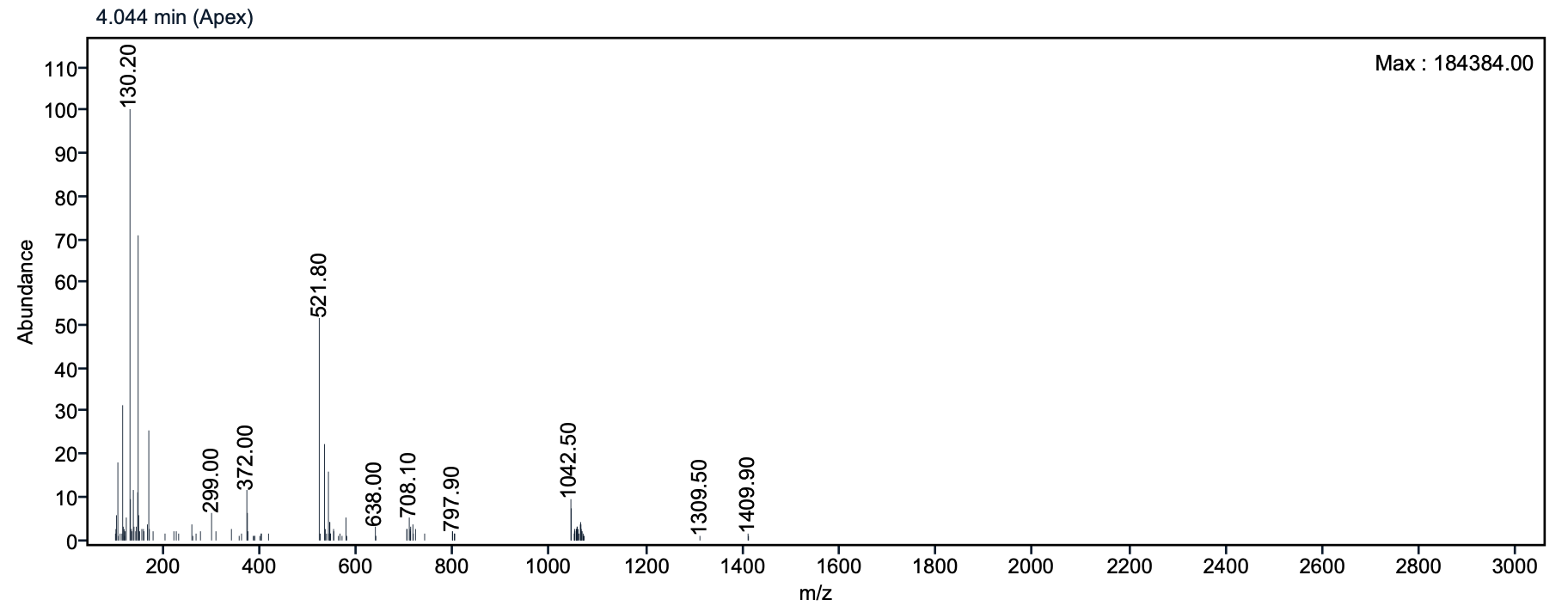
**

**Figure S7:** LC-MS spectrum of eFAP-7**.**


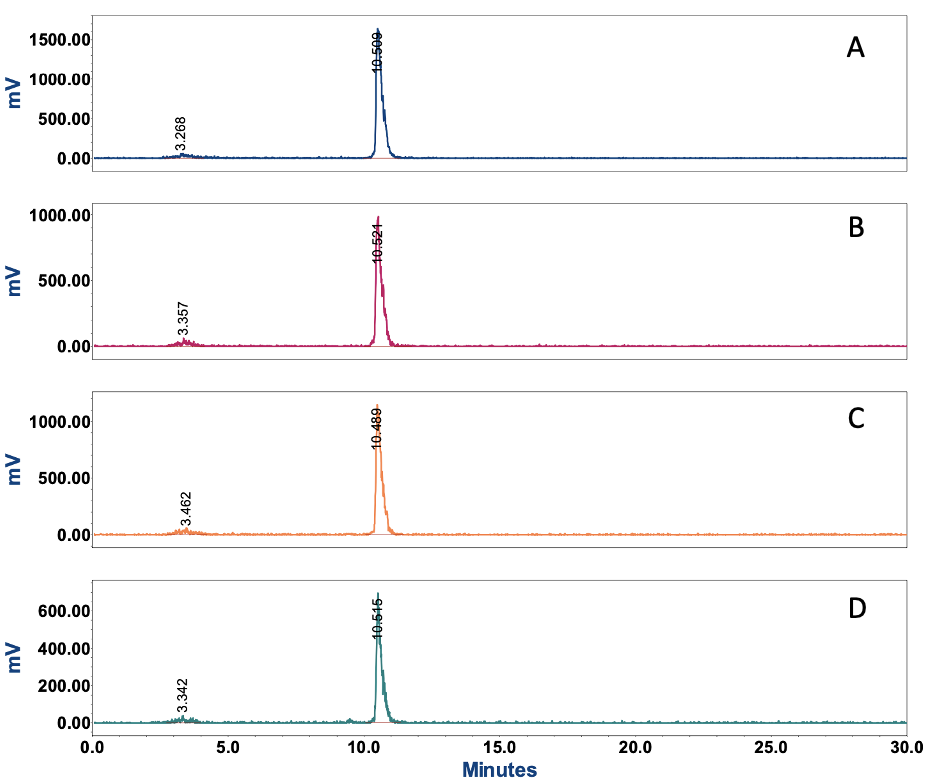


**Figure S8:** Radio-HPLC chromatograms of [^111^In]In-eFAP-6 (A) directly after labeling, (B) 1 h, (C) 4 h, and (D) 24 h after incubation in PBS at 37 °C.


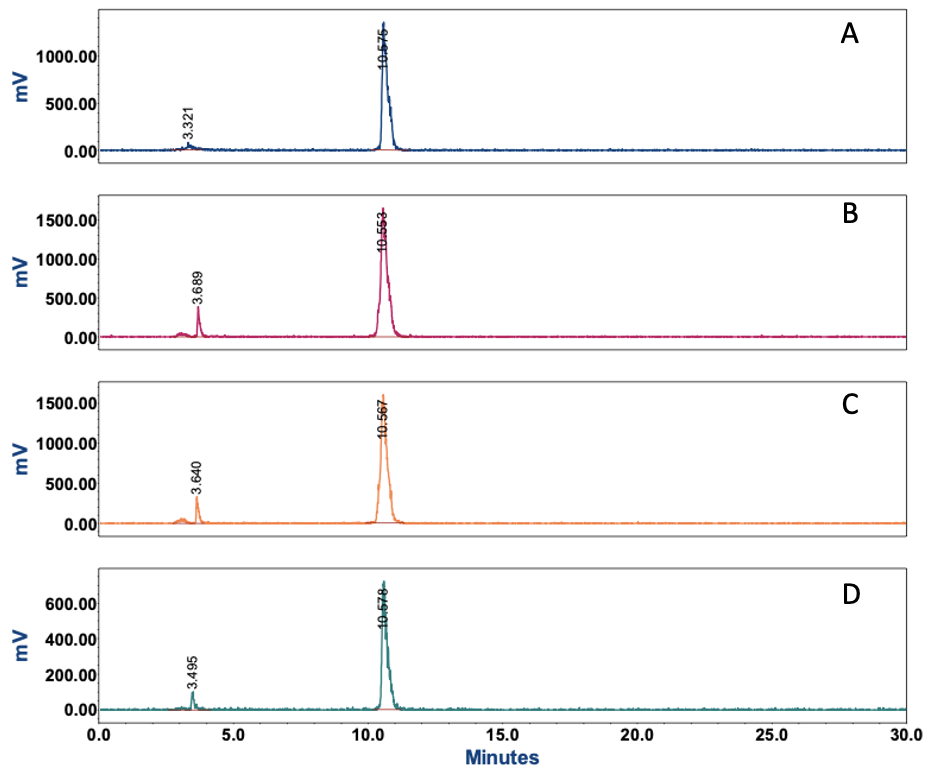


**Figure S9:** Radio-HPLC chromatograms of [^111^In]In-eFAP-6 (A) directly after labeling, (B) 1 h, (C) 4 h, and (D) 24 h after incubation in mouse serum at 37 °C.


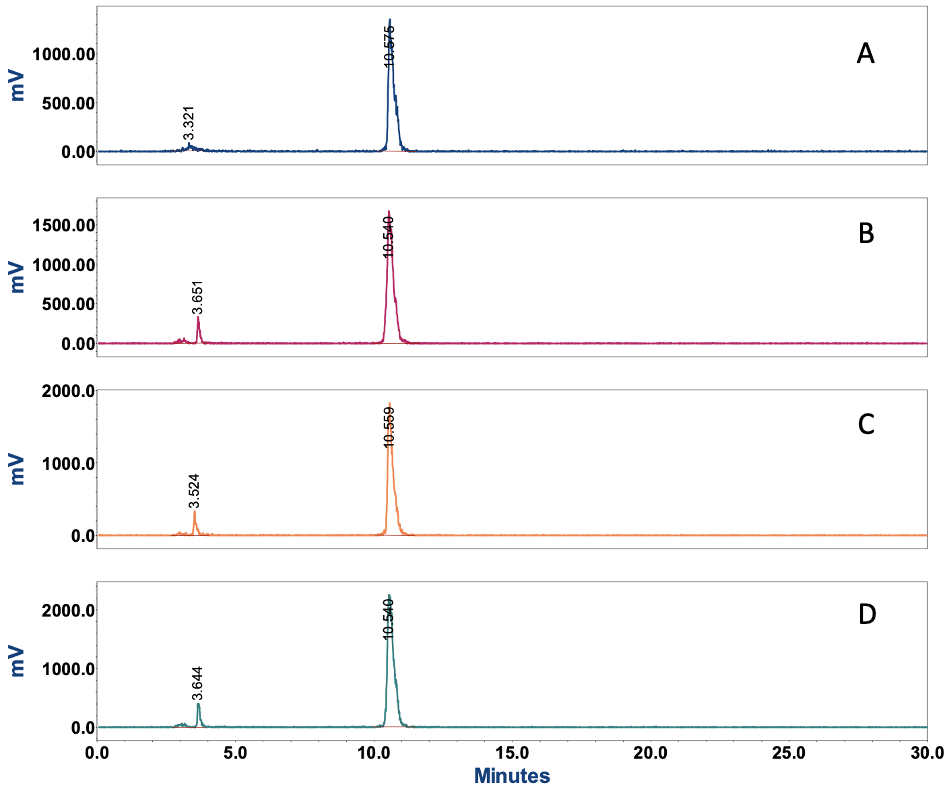


**Figure S10:** Radio-HPLC chromatograms of [^111^In]In-eFAP-6 (A) directly after labeling, (B) 1 h, (C) 4 h, and (D) 24 h after incubation in human serum at 37 °C.


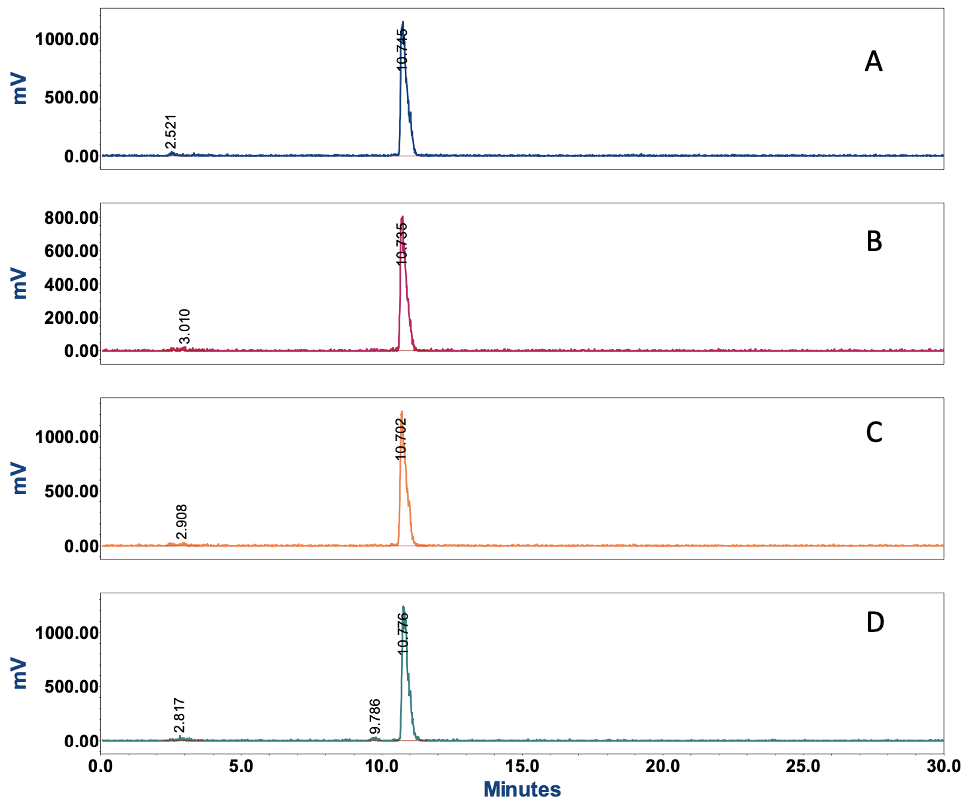


**Figure S11:** Radio-HPLC chromatograms of [^177^Lu]Lu-eFAP-6 (A) directly after labeling, (B) 1 h, (C) 4 h, and (D) 24 h after incubation in PBS at 37 °C.


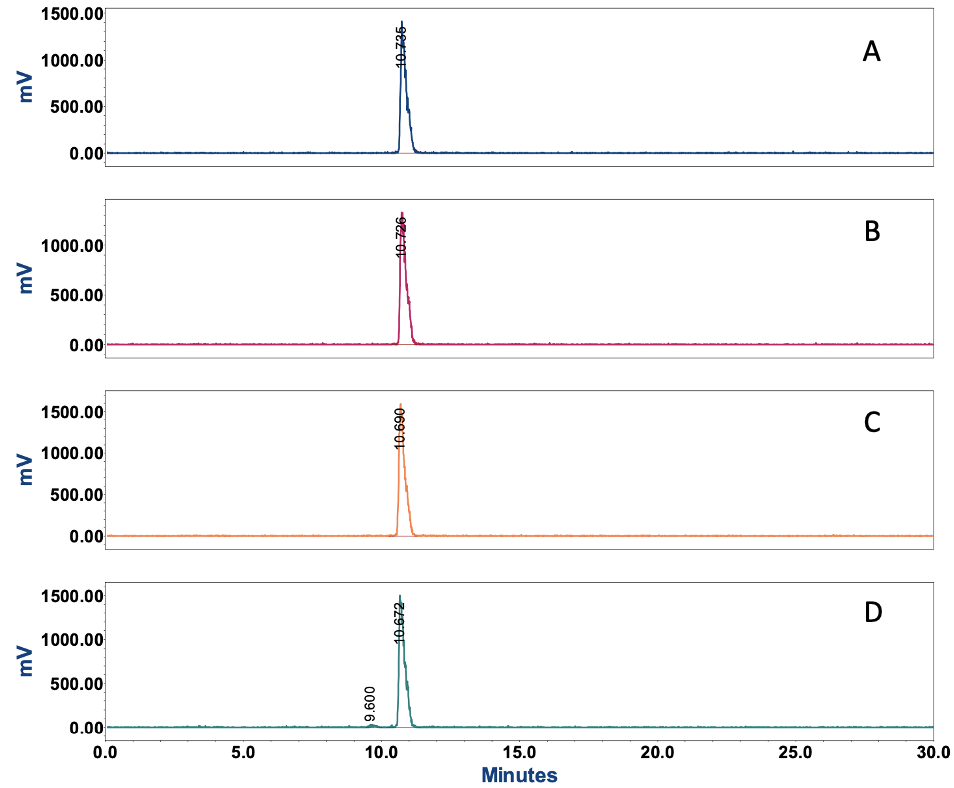


**Figure S12:** Radio-HPLC chromatograms of [^177^Lu]Lu-eFAP-6 (A) directly after labeling, (B) 1 h, (C) 4 h, and (D) 24 h after incubation in mouse serum at 37 °C.


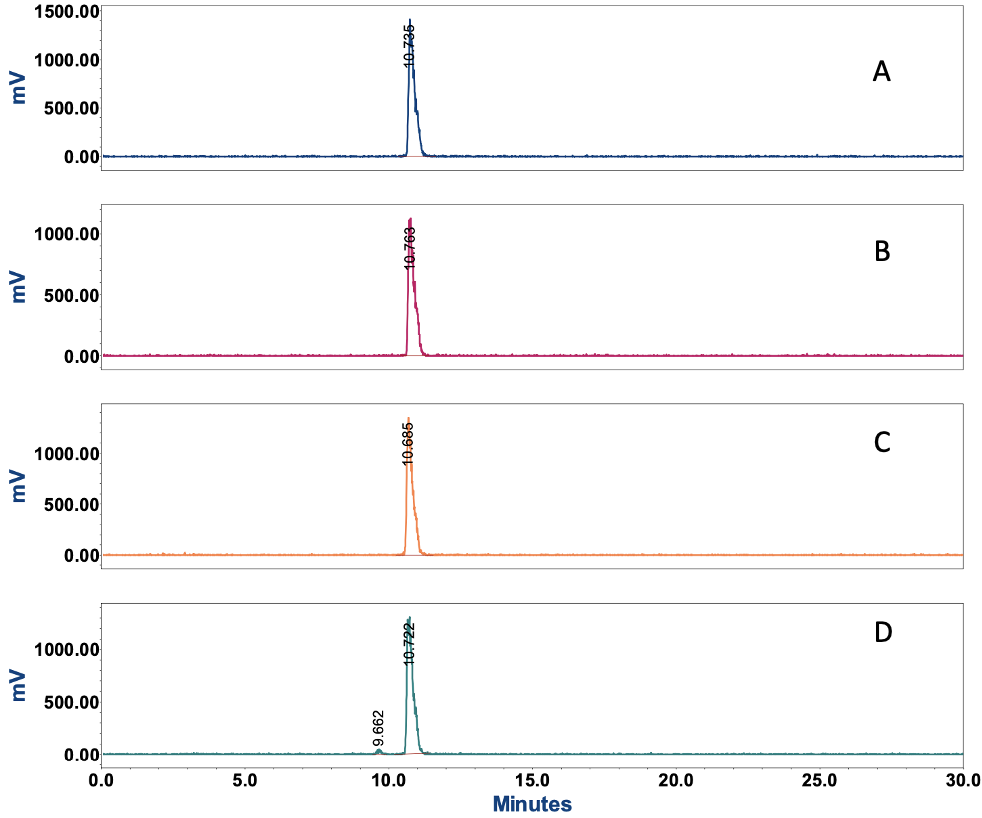


**Figure S13:** Radio-HPLC chromatograms of [^177^Lu]Lu-eFAP-6 (A) directly after labeling, (B) 1 h, (C) 4 h, and (D) 24 h after incubation in human serum at 37 °C.


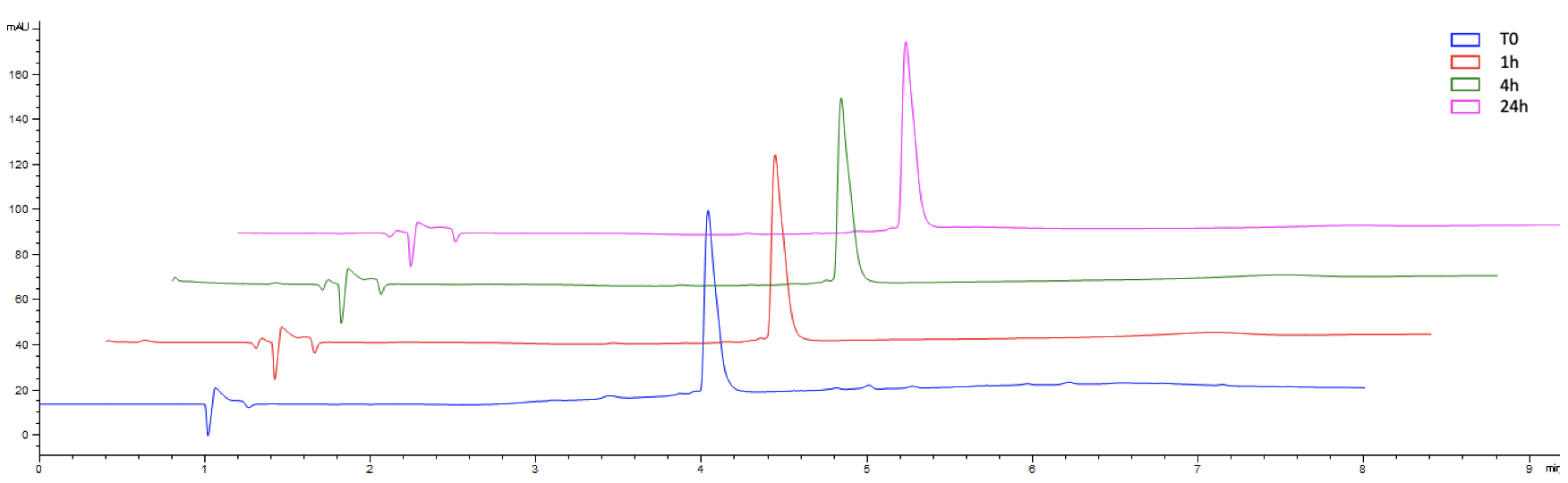


**Figure S14:** LC chromatograms of eFAP-7 directly after labeling (T0), 1 h, 4 h, and 24 h after incubation in PBS at 37 °C.


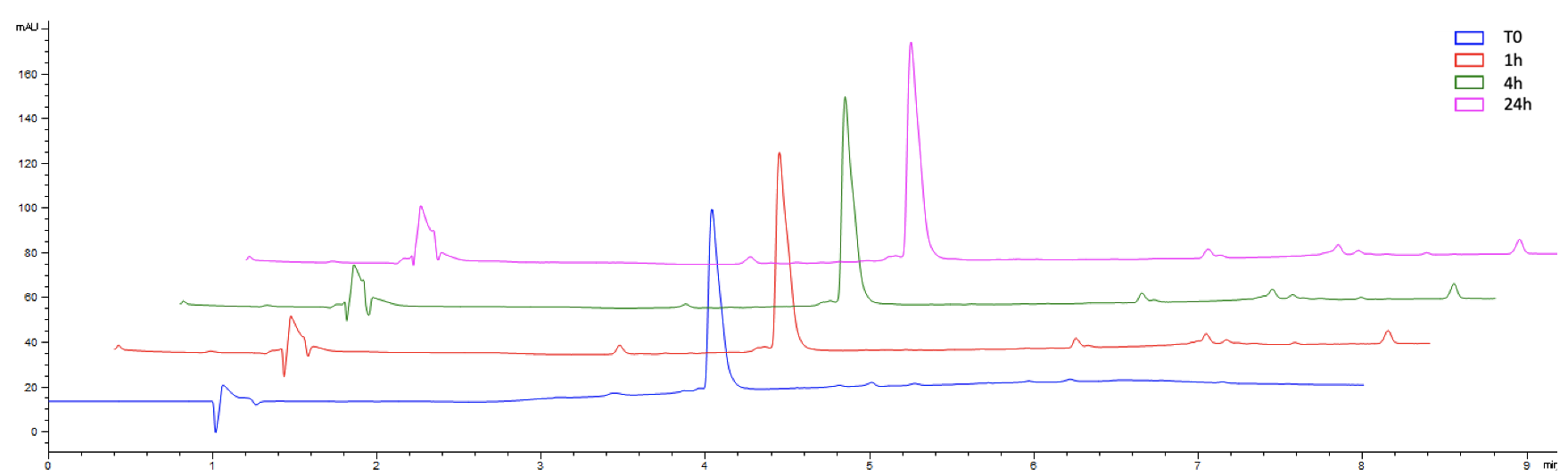


**Figure S15:** LC chromatograms of eFAP-7 directly after labeling (T0), 1 h, 4 h, and 24 h after incubation in mouse serum at 37 °C.


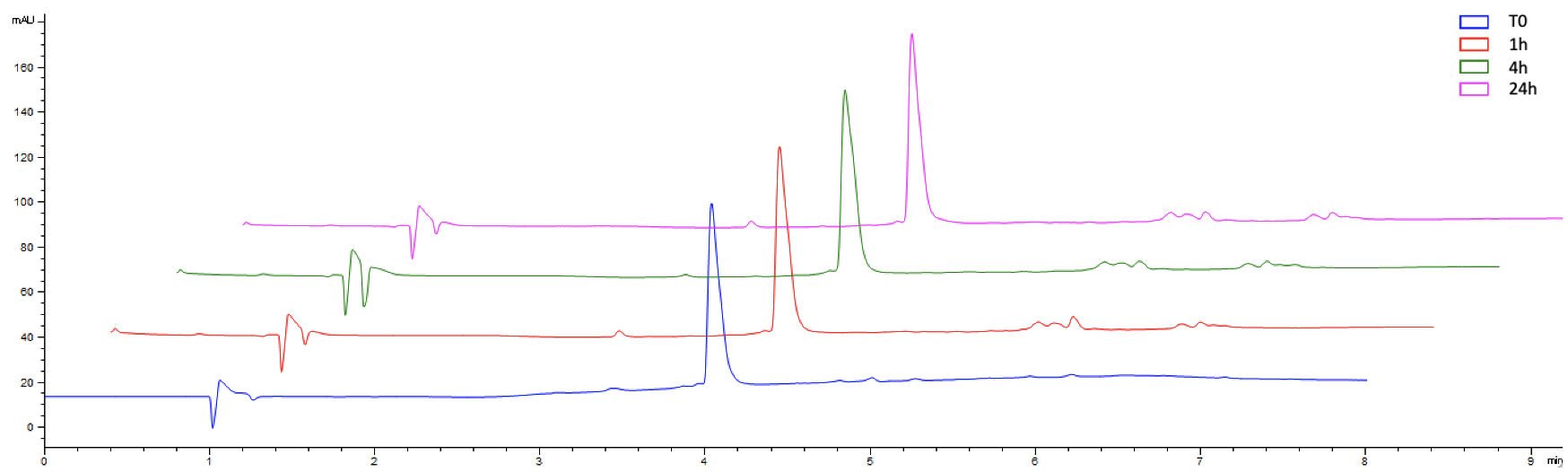


**Figure S16:** LC chromatograms of eFAP-7 directly after labeling (T0), 1 h, 4 h, and 24 h after incubation in human serum at 37 °C.


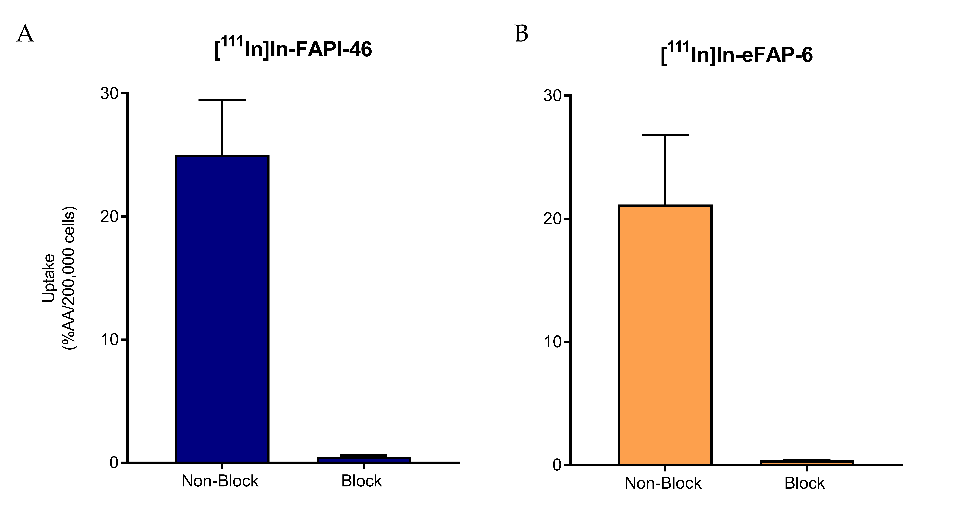


**Figure S17:**. In vitro uptake and blocked uptake experiment on HT1080-huFAP. Uptake of (A) [^111^In]In-FAPI-46 (n=3) and (B) [^111^In]In-eFAP6 (n=3) without (non-block) or with (block) simultaneous incubation of 1 mM UAMC-1110 after 45 min of incubation. Data are expressed as mean ± SD.


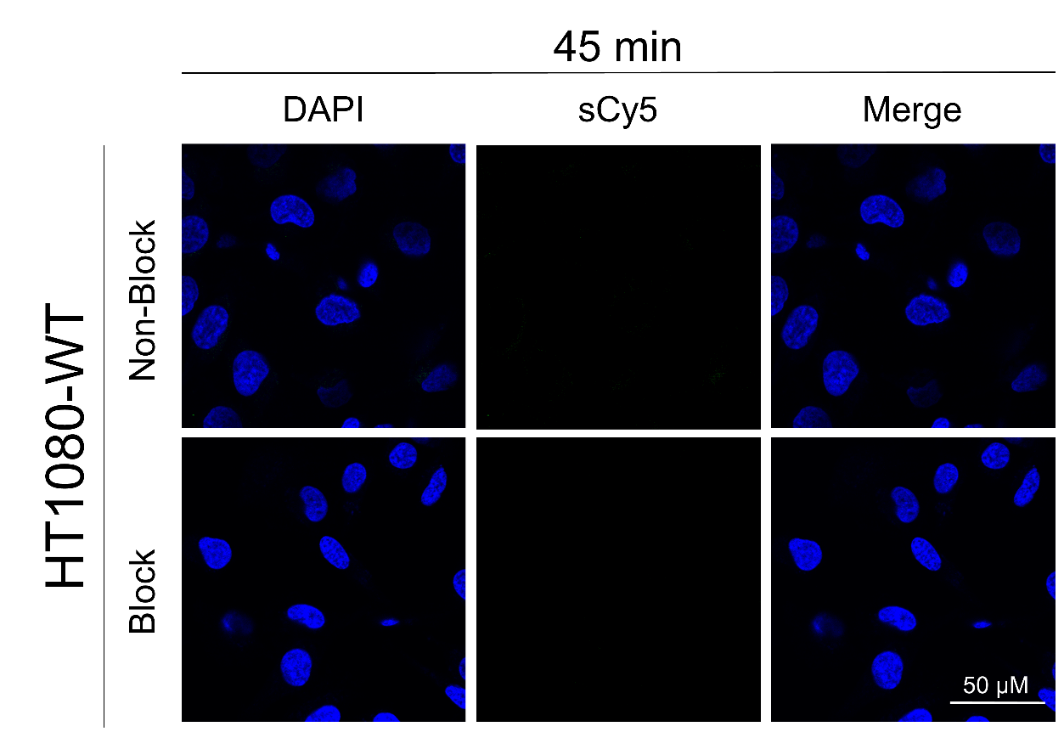


**Figure S18.** Confocal microscopy with HT1080-WT cells. Fluorescent uptake on HT1080-WT cells incubated with eFAP-7 and with (Block) or without (Non-Block) an excess of UAMC-1110 demonstrating the DAPI channel (blue), sulfo-Cyanine5 (sCy5) channel (green) and the overlay. Magnification is equal for all images. WT = wild-type

**Table S1.** Ex vivo biodistribution of [^177^Lu]Lu-FAPI-46 and [^177^Lu]Lu-eFAP-6 in HT1080-huFAP and HT1080-WT tumor-bearing mice. Data are expressed mean ± SD, as percentage injected dose per gram of tissue (%ID/g).

| [^177^Lu]Lu-FAPI-46 | | | [^177^Lu]Lu-eFAP-6 | | |  |
| --- | --- | --- | --- | --- | --- | --- |
| 1 h | **4 h** | **24 h** | **1 h** | **4 h** | **24 h** |  |
| Blood | 0,89 ± 0,13 | 0,37 ± 0,09 | 0,02 ± 0,01 | 0,38 ± 0,08 | 0,22 ± 0,07 | 0,01 ± 0,00 |
| Tumor  HT1080-huFAP | 4,95 ± 1,73 | 6,70 ± 0,85 | 1,88 ± 0,25 | 6,44 ± 1,09 | 5,92 ± 1,63 | 0,52 ± 0,10 |
| Tumor  HT1080-WT | 1,30 ± 0,42 | 0,54 ± 0,16 | 0,17 ± 0,08 | 0,50 ± 0,11 | 0,36 ± 0,11 | 0,06 ± 0,01 |
| Pancreas | 0,69 ± 0,23 | 0,29 ± 0,10 | 0,07 ± 0,03 | 0,25 ± 0,02 | 0,18 ± 0,06 | 0,01 ± 0,00 |
| Liver | 0,22 ± 0,02 | 0,26 ± 0,16 | 0,14 ± 0,04 | 2,32 ± 0,43 | 2,87 ± 1,18 | 1,67 ± 1,07 |
| Stomach | 0,28 ± 0,02 | 0,13 ± 0,03 | 0,03 ± 0,01 | 0,33 ± 0,32 | 0,11 ± 0,04 | 0,02 ± 0,01 |
| Small intestines | 0,24 ± 0,06 | 0,12 ± 0,01 | 0,02 ± 0,01 | 0,33 ± 0,34 | 0,12 ± 0,04 | 0,02 ± 0,00 |
| Cecum | 0,76 ± 0,14 | 0,40 ± 0,09 | 0,07 ± 0,02 | 0,36 ± 0,01 | 0,32 ± 0,08 | 0,03 ± 0,00 |
| Colon | 0,29 ± 0,02 | 0,18 ± 0,06 | 0,04 ± 0,01 | 0,19 ± 0,05 | 0,14 ± 0,03 | 0,02 ± 0,01 |
| GI Tract^a^ | 0,42 ± 0,04 | 0,22 ± 0,01 | 0,04 ± 0,01 | 0,69 ± 0,92 | 0,17 ± 0,05 | 0,02 ± 0,00 |
| Kidneys | 1,41 ± 0,30 | 1,14 ± 0,18 | 0,17 ± 0,05 | 2,46 ± 0,83 | 1,11 ± 0,21 | 0,37 ± 0,09 |
| Lungs | 0,53 ± 0,10 | 0,19 ± 0,04 | 0,03 ± 0,01 | 0,33 ± 0,08 | 0,15 ± 0,03 | 0,01 ± 0,00 |
| Heart | 0,36 ± 0,05 | 0,17 ± 0,05 | 0,04 ± 0,02 | 0,15 ± 0,04 | 0,10 ± 0,03 | 0,01 ± 0,00 |
| Salivary glands | 1,63 ± 0,32 | 0,71 ± 0,25 | 0,14 ± 0,05 | 0,56 ± 0,08 | 0,41 ± 0,14 | 0,02 ± 0,00 |
| Muscle | 0,66 ± 0,38 | 0,26 ± 0,10 | 0,06 ± 0,03 | 0,19 ± 0,03 | 0,16 ± 0,06 | 0,01 ± 0,00 |
| Bone | 3,04 ± 1,10 | 1,56 ± 0,28 | 0,50 ± 0,08 | 1,80 ± 0,44 | 1,27 ± 0,24 | 0,19 ± 0,01 |

^a^The GI-tract is the uptake of the stomach, small intestines, cecum and colon combined.

GI-tract = gastro-intestinal tract, huFAP = human fibroblast activation protein, WT = wild-type
